# Nanoparticles—Attractive Carriers of Antimicrobial Essential Oils

**DOI:** 10.3390/antibiotics11010108

**Published:** 2022-01-14

**Authors:** Arya Nair, Rashmi Mallya, Vasanti Suvarna, Tabassum Asif Khan, Munira Momin, Abdelwahab Omri

**Affiliations:** 1 Department of Quality Assurance, SVKM’s Dr. Bhanuben Nanavati College of Pharmacy, Mumbai 400056, Maharashtra, India; arya.h.nair@gmail.com (A.N.); rashmi.mallya@bncp.ac.in (R.M.); 2 Department of Pharmaceutical Chemistry & Quality Assurance, SVKM’s Dr. Bhanuben Nanavati College of Pharmacy, Mumbai 400056, Maharashtra, India; vasanti.suvarna@bncp.ac.in (V.S.); tabassum.khan@bncp.ac.in (T.A.K.); 3 Department of Pharmaceutics, SVKM’s Dr. Bhanuben Nanavati College of Pharmacy, Mumbai 400056, Maharashtra, India; munira.momin@bncp.ac.in; 4 The Novel Drug & Vaccine Delivery Systems Facility, Department of Chemistry and Biochemistry, Laurentian University, Sudbury, ON P3E 2C6, Canada

**Keywords:** essential oils, nanoparticles, antimicrobials

## Abstract

Microbial pathogens are the most prevalent cause of chronic infections and fatalities around the world. Antimicrobial agents including antibiotics have been frequently utilized in the treatment of infections due to their exceptional outcomes. However, their widespread use has resulted in the emergence of multidrug-resistant strains of bacteria, fungi, viruses, and parasites. Furthermore, due to inherent resistance to antimicrobial drugs and the host defence system, the advent of new infectious diseases, chronic infections, and the occurrence of biofilms pose a tougher challenge to the current treatment line. Essential oils (EOs) and their biologically and structurally diverse constituents provide a distinctive, inexhaustible, and novel source of antibacterial, antiviral, antifungal, and antiparasitic agents. However, due to their volatile nature, chemical susceptibility, and poor solubility, their development as antimicrobials is limited. Nanoparticles composed of biodegradable polymeric and inorganic materials have been studied extensively to overcome these limitations. Nanoparticles are being investigated as nanocarriers for antimicrobial delivery, antimicrobial coatings for food products, implantable devices, and medicinal materials in dressings and packaging materials due to their intrinsic capacity to overcome microbial resistance. Essential oil-loaded nanoparticles may offer the potential benefits of synergism in antimicrobial activity, high loading capacity, increased solubility, decreased volatility, chemical stability, and enhancement of the bioavailability and shelf life of EOs and their constituents. This review focuses on the potentiation of the antimicrobial activity of essential oils and their constituents in nanoparticulate delivery systems for a wide range of applications, such as food preservation, packaging, and alternative treatments for infectious diseases.

## 1. Introduction

Microorganism-caused infections are a source of concern for public health. Overuse or underuse of antimicrobials has resulted in the global rise of multidrug-resistance in microorganisms, including bacteria, fungi, viruses, parasites, and protozoans. Every year, more than two million people suffer from infections with antimicrobial resistance and by the year 2050, the annual global mortality rate of these infections is expected to reach 10 million [1]. Antimicrobial resistance develops and continues to transmit across different species of bacteria due to various factors such as conjugation, transformation, and transduction processes of the gene transfer cycle. Therefore, new and unique alternative antimicrobials are needed to combat multidrug resistance [2,3,4].

Essential oils are aromatic liquids produced through a series of complex metabolic pathways in plants with the goal of defending them from a wide range of pathogens and are commonly extracted by steam distillation [4,5]. Different factors influencing the chemical compositions of EOs include the species, climatic conditions, soil condition, fertilization, genotype, mode of production, harvest seasons, and extraction procedure. Two major groups of chemical compounds present in EOs are (i) aromatic and aliphatic compounds, and (ii) hydrocarbon terpenes (isoprenes) and terpenoids (isoprenoids). Terpenes are five-carbon isoprene units (C_5_H_8_) that constitute the largest class of chemical compounds present in essential oils. Terpenes are categorized as mono-, sesqui-, di-, ses-, tri-, and tetra-terpenes or alternate hemi-terpenes based on the number of carbon atoms present in the structure. However, the monoterpenes and sesquiterpenes are the most important constituents of essential oils responsible for their characteristic aroma of EOs. Monoterpenes are composed of two isoprene units and exist in monocyclic, bicyclic, and acyclic forms, whereas sesquiterpenes are made up of three isoprene units and occur in acyclic, monocyclic, bicyclic, and tricyclic forms. Chemical modification of a terpene or sesquiterpene, through oxidation or structural rearrangement, produces different terpenoids. Thus, EOs with diverse chemical compositions exhibit a wide range of therapeutic effects [6,7,8].

### 1.1. Mechanism of Action and Bacterial Spectrum

Essential oils have been widely explored on a large scale as potential sources of novel antimicrobial agents, food preservatives, and alternative treatments for infectious diseases due to their antifungal, antiparasitic, antibacterial, and antiviral properties (Table 1) [4]. The antimicrobial mechanism of action varies with the type of EO or the strain of the microorganism used. Gram-negative bacteria have a thick lipopolysaccharide layer which reduces the susceptibility of microorganisms towards EOs but gram-positive bacteria will lack this lipopolysaccharide. Hence, EOs can enter gram-positive bacteria easily as compared to gram-negative bacteria. Due to the presence of lipoteichoic acid, the entry of EOs into gram-positive microbial cells is eased. Various research investigations have demonstrated that the bioactive components contained in EOs attach to the cell surface and penetrate the phospholipid bilayer of the cell membrane, followed by membrane damage, which causes negative impacts on cell metabolic activities and cell death. Alteration of the cell membrane integrity results in the loss of important intracellular components such as proteins, reducing sugars, ATP, and DNA, and also blocks ATP synthesis and associated enzymes, resulting in electrolyte leakage and cell death. At the minimum inhibitory concentration (MIC), it was found that the EOs damaged the bacterial cell membrane. However, at the minimum bactericidal concentration (MBC), the EOs destroyed the bacterial cells [3,4].

**Figure 1:**
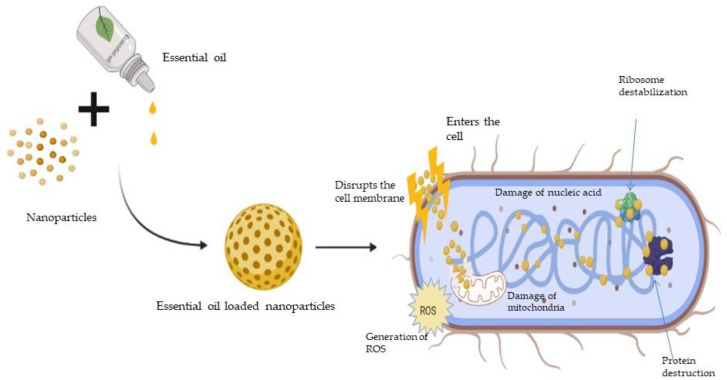
Mechanism of action of antimicrobial activity of essential oils.

The primary methods of action of antimicrobial drugs are categorised. Interference with cell wall biosynthesis (β-lactams and glycopeptides agents), inhibition of bacterial protein synthesis (macrolides and tetracyclines), interference with nucleic acid synthesis (fluroquinolones and rifampin), inhibition of a metabolic pathway (trimethoprim-sulfamethoxazole), and disruption of bacterial membrane structure (polymyxins and daptomycin) are all examples of these mechanisms [9]. The biosynthesis of cell walls, proteins, and nucleic acids are three of the principal targets for antibiotics. Bacteria have developed diverse resistances to antibiotics over the years in order to survive the flood of antibiotics. The processes differ, making the task of preventing resistance spread more difficult. As the threat of medication resistance grows, researchers are turning their attention to natural materials with antimicrobial capabilities, such as plants, as a potential supply of antimicrobial medicines in the future. Various mechanisms of the antimicrobial action of essential oils is depicted in Figure 1.

*Bunium persicum* and *Homalomena pineodora* oil and its constituents such as γ-Terpinene, 1-phellandrene, γ-terpene, and cuminaldehyde exert antimicrobial action by cell membrane disruption, cytolytic leakage and swelling, and the reduction in membrane function [10]. *Cananga odorata, Citrus bergamia*, *Cymbopogon citratus*, *Citrus reticulata*, *Lavandula angustifolia Sevastopolis*, *Rosmarinus officinalis*, and *Ocimum basilicum* essential oils contain linalool, citral, borneol, camphor, and linalyl acetate that exhibit antimicrobial activity through the disruption of cell membrane integrity and induce changes in ATP concentration and cell membrane hyperpolarization, as well as reducing cytoplasmic pH [11]. *Carum copticum*, *Cinnamomum zeylanicum*, *Lippia sidoides*, *Mentha piperita*, *Origanum vulgare*, *Thymus vulgaris*, and *Zataria multiflora* essential oils are reported to consist of thymol, carvacrol, p-cymene, menthol, and menthone, and display antimicrobial effects through depolarization of the cytoplasmic membrane and disruption of the cell membrane integrity as well as the decreasing of intracellular ATP levels [12]. *Eugenia caryophyllata*, *Eucalyptus globulus*, *Pistacia atlantica*, *Pistacia lentiscus*, and *Punica granatum* essential oil and their constituents such as *β*-caryophyllene, Eugenol, α-Pinene, β-Pinene, β-myrcene, and 1,8-cineole exhibit antimicrobial activity by apoptosis via nuclear condensation and fragmentation pathways, including the disruption of mitochondrial membrane potential [13].

Components of essential oils (mostly with phenolic structures) were able to display a broad spectrum of antibacterial activity, indicating that the chemical structures of the components have a significant impact on their effectiveness and manner of antibacterial action [9]. To understand the efficiency of EOs in comparison to antibiotics Gavanji et al. evaluated the antibacterial activity of *Artemisia kermanensis*, *Lavandula officinalis*, *and Zataria multiflora* Boiss essential oils against *Staphylococcus aureus*, *Pseudomonas aeruginosa*, and *Klebsiella pneumoniae* with ampicillin, penicillin, and tetracycline as positive control antibiotics. Different concentrations of essential oils (0.08–100 µg/disk) were used and the results showed that the concentration of 100 µg/disk of each of the three essential oils was more efficient compared with lower concentrations on the bacteria. A comparison between the three plant essential oils (at a concentration of 100 µg/disk) and positive control antibiotics (ampicillin, penicillin, and tetracycline of 10 µg, 10 µg and 30 µg) demonstrated that *Z. multiflora* Boiss essential oil (at 24 h, 48 h and 72 h) exhibited a stronger antibacterial effect (bigger inhibition zone) against *S. aureus* (27.80 ± 0.20, 28.67 ± 0.33, 28.67 ± 0.33), *K. pneumonia* (27.83 ± 0.12, 28.10 ± 0.21, 28.10 ± 0.21) and *P. aeruginosa* (19.90 ± 0.27, 20.40 ± 0.23, 20.53 ± 0.18) respectively. Since bacteria are becoming increasingly resistant to antibiotics, employing these essential oils as natural and alternative antibacterial compounds may be beneficial. Some other examples of essential oils or plant extracts commonly used for their antimicrobial properties are tea tree oil, ylang-ylang, betel pepper, manuka, eucalyptus, arnica, lemon verbena, rosemary, green tea extract, and calendula. Although extensively practiced since ancient times, the use of natural extracts from plants as antimicrobial compounds declined after the development of synthetic antibiotics [14]. Duarte et al. demonstrated that EOs with MIC values of up to 0.5 mg/mL have strong antibacterial action, EOs with MIC values between 0.6 and 1.5 mg/mL have moderate antimicrobial activity, and EOs with MIC values over 1.6 mg/mL have weak antimicrobial activity [15]. Essential oils showed activity against *Helicobacter pylori* in the MIC range of 20–589 µg/mL and demonstrated activity against bacteria most frequently isolated from the respiratory tract including *Streptococcus pneumoniae, Haemophilus influenzae, Moraxella catarrhalis, and Streptococcus pyogenes* at the MIC range of 1.56–6.25 µg/mL. Monoterpene alcohols and aldehydes, as well as phenols and cinnamaldehyde, were the most active ingredients, with MIC-values of 160–300 µg/mL against both *S. pneumoniae and H. influenzae.* In vitro cytotoxicity studies of various EOs such as lavender oil, lemon oil, clove oil, thyme oil, and mentha oil on different cell lines such as HMEC-1 (microvascular endothelial cells), HNDF (dermal fibroblasts), 153BR (fibroblasts), and RC-37 demonstrated an effective concentration (cytotoxic to 50% of the tested cells) range of 5–1950 µg/mL [16].

The antimicrobial potential of EOs is determined by the spectrum of microbial targets it affects. Essential oils obtained from clove, cinnamon, oregano, pimento, rosemary, and thyme demonstrated strong antibacterial activity against *Staphylococcus aureus*, *Salmonella typhi*, and *Pseudomonas aeruginosa*.

- Clove essential oil demonstrated in vitro inhibitory and bactericidal activity at a concentration of 0.304 mg/mL against *S. aureus, Escherichia coli, Listeria monocytogenes*, and *Salmonella typhimurium* [17]. The antiviral activity of eugenol, the primary component of clove essential oil, was investigated in vitro against the Herpes simplex virus (HSV)-1 and HSV-2 viruses. The replication of these viruses was inhibited with IC_50_ values of 25.6 µg/mL and 16.2 µg/mL against HSV-1 and HSV-2, respectively [16]. The MIC value of clove oil against *L. monocytogenes* was found to be 0.5 mg/mL [18].
- Lavender EO obtained from *L. angustifolia* Mill. has a strong antiseptic effect against antibiotic-resistant strains, e.g., *Staphylococcus aureus*, that are resistant to methicillin (MRSA) or vancomycin-resistant strains of *Enterococcus* sp. (VRE). The antimicrobial activity of Lavender EO was evaluated against *L. monocytogenes* (24 strains) and *Salmonella enterica* (10 food strains). MIC ≥ 10.0 μL/mL inhibited *Salmonella*; MIC of 0.3 μL/mL inhibited *L. monocytogenes*, revealing noticeable activity, especially on clinical strains. This activity appears to be related to EOs composition. The highest antimicrobial activities were demonstrated in the specific constituents such as linalool (38.17 and 61.98%), camphor (8.97 and 10.30%), and 1,8-cineole (6.89 and 8.11%, respectively) [19].
- Thyme EO was found to have antiviral action against Herpes simplex virus (HSV1, DNA virus) with IC_50_ values of 11 µg/mL [19]. Thyme EO was also tested for its ability to fight strains that cause acute bacterial pharyngitis and throat irritation. *β-haemolytic Streptococci* strains, such as *S. pyogenes*, cause this infection. *T. vulgaris* EO was found to be effective against *S. pyogenes* strains obtained from throat of patients [20]. At a concentration of 0.06%, thyme EO that was rich in γ-terpinene (68.415%) and p-thymol (24.721%) totally inhibited the growth of *Fusarium graminearum* Fg 06–17 [21].
- Essential Oil of *Cinnamomum zeylanicum* demonstrated 100% inhibition effect at 3.1 µL/mL concentration against influenza virus A1/Denver/1/57 (H1N1) with 30 min exposure. In both liquid and vapour phases, Eugenol, the main component of *Cinnamomum zeylanicum* EO, exhibited the most significant anti-influenza activity [22]. Cinnamon essential oil was recently used to improve zein film for food packaging, which now contains an extra 4% concentration of chitosan nanoparticles (CNP). The combined antibacterial capabilities of EO and nanoparticles not only inhibited the development of *Escherichia coli* (PTCC 1163) and *Staphylococcus aureus* (PTTC 25923), but also increased the tensile strength and decreased the elongation of the composite zein film [23].
- Tea tree EO has been used in products for oral hygiene and dermatological uses due to its antibacterial characteristics. *Porphyromonas gingivalis* (MIC and MBC = 0.007%) and *Porphyromonas endodontalis* (MIC = 0.007% and MBC = 0.5%) bacteria that cause halitosis are both inhibited by tea tree EO [24]. The antibacterial activities of tea tree essential oils (EOs) that are commercially accessible were examined. Five out of the ten EOs were active. Components identified in tea tree essential oil inhibited bacterium viability in *Pseudomonas aeruginosa* biofilm and caused oxidative damage in *Candida glabrata* [25]. Essential oil of *Melaleuca alternifolia*, on the other hand, displayed only minimal antifungal activity against *Aspergillus niger* (MIC = 625 µg/mL), which was attributed to the active components terpinen-4-ol and α-terpineol [26].

### 1.2. Stability and Bioavailability of Essential Oils

Essential Oils are susceptible to degradation due to external factors such as light, temperature, oxidation, or hydrolysis. The final composition of EOs depends on chemical composition of EOs, plant material processing and storage, distillation methods, and subsequent storage of EOs. The chemical constituents of EOs have a significant impact on its stability. Double bonds present in the constituents undergo autoxidation as hydrogen atom abstraction leads to resonance-stabilized radicals. Conjugated double-bonds can stabilize radicals formed by polyunsaturated terpene hydrocarbons. Simultaneously, isomerization to tertiary radicals might occur, resulting in oxidative degradation. The presence of aerial oxygen triggers spontaneous free radical chain reactions, which result in the formation of unstable hydroperoxides that breakdown in the presence of light, heat, or rising acidity. Stable secondary oxidation products include monovalent to polyvalent alcohols, aldehydes, ketones, epoxides, peroxides, acids, or oxygen-bearing polymers. Some terpenoids transform into oxidized secondary products without the creation of hydroperoxides. Since headspace oxygen diffuses into the sample over time, the EOs should be maintained in completely filled containers or, if possible, treated with inert gas to remove any leftover air and prevent oxidative reactions. Light and temperature are the other two elements that are strongly linked to EO oxidative degradation. Light enhances autoxidation and the generation of alkyl radicals in monoterpenes, catalyzes intramolecular isomerization events or trans–cis conversions, and boosts monoterpene degradation. Heat speeds up chemical reactions and aids in the development of primary auto-oxidation products, such as hydroperoxides, which are then degraded when the temperature rises, yielding final oxidation products. At high temperatures, volatiles are thermolabile and vulnerable to rearrangement processes. Cleavage of double bonds, epoxidation, dehydrogenation into aromatic systems, and allylic oxidation into alcohols, ketones, and aldehydes are the four types of oxidative processes that occur during the thermal breakdown of terpenes. The production of alkyl or hydroxyl radicals is more apparent at higher temperatures because oxygen solubility is lower whereas storing EOs at low temperatures promotes oxygen solubility in liquids, resulting in the formation of peroxide. Compounds, primarily isoprenoids, easily oxidize in complex mixtures such as EOs, causing rearrangement and breakdown processes in more stable structures. In exchange, phenylpropanoids found in essential oils work as antioxidants, scavenging free radicals and protecting other molecules from degradation. Essential Oils are losing their quality as a result of the decomposition mechanisms detailed above. Changes in colour, consistency, and odour are the most visible indications of age, with the latter being particularly unpleasant and smelly. The biological activity of EOs is significantly influenced by their general physicochemical characteristics (complexity and interactions of individual compounds) and constituents (low molecular weight, presence of diverse functional groups in the molecule, reactivity, and hydrophobicity) [27].

The bioavailability and bioaccessibility of plant metabolites, including EOs and their individual terpene compounds, are analyzed using various in vivo and in vitro methods. Bioaccessibility is often estimated using in vitro digestive models. The majority of these approaches work by altering the pH and introducing certain digestive enzymes to simulate the conditions of gastrointestinal (GI) system. In vivo animal and clinical research have also been used to investigate bioavailability. Physiochemical, biochemical, and physiological interactions all have an impact on the bioavailability of EOs. It is considered that intravenous administration of EOs has the highest bioavailability (100%) and that other administration routes have lower bioavailability. However, as observed for 1,8-cineole, which has a bioavailability rate of 95.6%, the bioavailability of EO components administered orally may be very high. Nonetheless, recent studies show that most EOs are readily absorbed when applied topically, orally, or through the lungs. Most EO compounds are known to penetrate from the surface of skin, through the stratum corneum, into the dermis, and finally into the bloodstream. The high percutaneous absorption rates of EOs should be included in systemic toxicity risk assessments due to their lipophilic properties. The beneficial effects of essential oils on the respiratory system when inhaled are well-known. For large systemic concentrations required for bioactivity in the colon, rectal suppositories are employed. However, due of the great sensitivity of the rectal mucosal membrane to EOs and the potential for irritation, dosages and concentrations should be carefully controlled [28].

Essential oils have a wide range of therapeutic properties; however, their use as antimicrobials in alternative treatments for infectious diseases, food preservation, and packaging has been restricted due to issues such as low solubility, solvent toxicity, volatility, and strong organoleptic flavor. Scientific investigations focusing on various encapsulation strategies for EOs such as solid lipid nanoparticles, inorganic nanoparticles, polymeric nanoparticles, nanogels, liposomes, silica nanoparticles, and metallic nanoparticles are directed towards masking their undesirable attributes and enhancing their biological activities [2].

## 2. Essential Oils in Combination with Antibiotics

Many approaches are investigated to resolve the antimicrobial resistance crisis. The creation of antibiotic alternatives, as well as the discovery or development of adjuvants, are among the potential options explored. The current state of knowledge on the modes of action of EO elements and their synergy with antibiotics is provided, as well as proposed pathways by which they interact (Table 2). To improve antibiotic efficacy, researchers must identify ways to improve drug diffusion through bacterial membranes and/or to inhibit efflux pumps, which are a common resistance mechanism in gram-negative bacteria. A proposed specific target for EO components is the inhibition of efflux pumps, responsible for antibiotic resistance. Hence, EOs can be used in combination with antibiotics. The checkerboard assay with Fractional Inhibitory Concentration Index (FICI) computation is the most commonly reported assay method [72].

## 3. Clinical Trials and Marketed Products of EOs

Several clinical studies of EOs and their formulations, which evaluated their antibacterial efficacy, are reported (Table 3). The beneficial characteristics of essential oils, particularly their antimicrobial properties and their diversity in products and manufacturing techniques, in addition to greater microbial diversity, have positioned EO-based formulations as subjects of interest in recent investigations.

### Marketed Products

The compound annual growth rate of the global essential oil market is expected to rise to 8.6% from 2019 to 2025. According to the recent reports by Global Market Insights, Inc, the worldwide essential oil market will cross USD 13 billion in the year of 2024. Firmenich, Frutarom, Flaex, Rock Mountain Moksha Lifestyle, and Florihana Falcon Young Living are the prominent firms with a large market share of essential oil-based products. Worldwide, EOs are used in a large number of products, and about 300 types of essential oils out of 3000 are commercially significant in various kinds of industries including cosmetics, foods, beverages, agronomics, perfumes, sanitary products, and pharmaceuticals. Essential oil constituents, including limonene, geranyl acetate, carvone, etc., are important components of hygienic products such as mouthwashes, bodywashes, cleansers, and toothpastes. Essential oils are extensively used in personal care products such as creams, lotions, facemasks, facewashes, bath soaps, and aromatherapy items as massage and inhalation aids, and in paint and plastic products, textiles, and pharmaceutical formulations as masking agents to avoid unpleasant odours. Essential oils are extensively used in various kinds of cereals, in the antimicrobial packing of food items, in edible thin films, in the preservation of fruits, vegetables, seafoods, and soft drinks, and as flavouring agents in carbonated drinks, and as major ingredients in soda/citrus concentrates, etc. [96]. Essential oils enhance the dermato-cosmetic properties of formulations through their antimicrobial and preservation properties in addition to antiacne, anti-inflammatory, antiaging, skin lightening, and sun protection properties. Commonly used essential oils for cosmetic applications are lavender oil, frankincense oil, tea tree oil, cedar wood oil, rosemary oil, and grapefruit oil [97]. Lavender oil, clary sage oil, peppermint oil, rosemary oil, cinnamon oil, lemon oil, eucalyptus oil, bergamot oil, lemon grass oil, ylang-ylang oil, sandalwood oil, chamomile oil, jasmine oil, and grapefruit oil are some of the essential oils used in aromatherapy [97]. Essential oils are widely used in the active packaging industry to extend shelf life, assure product quality and safety, and improve product appearance. The incorporation of essential oils to films increases their water vapour barrier properties and provides antibacterial and antioxidant properties. Jasmine, rosemary, peppermint, cinnamon, oregano, thyme, cumin, eucalyptus, rosewood, clove, tea tree, palmarosa, geranium, lavender, lemongrass, mandarin, bergamot, and lemon are some of the most commonly utilised EOs in packaging systems. Different food products such as fresh beef, butter, fresh octopus, ham, and fish are available in the food matrices on which packaging solutions containing EOs are applied [97].

## 4. Nanotechnology in Delivery of Essential Oils

**Figure 2:**
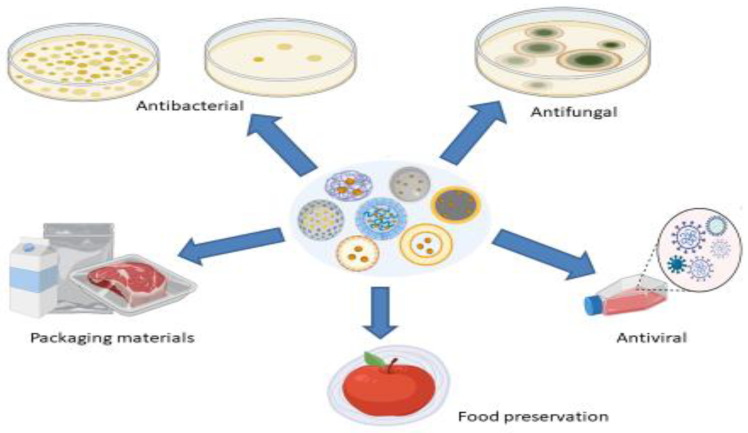
Application of nanoparticles.

Nanoparticles are particles in the size range of 1 to 100 nm, with one or more dimensions, with improved properties compared to corresponding materials of higher sizes, such as high reactivity, sensitivity, surface area, stability, and strength. A variety of nanomaterials such as gold, silver, platinum, iron, copper, chitosan, and zinc have been utilized in the fabrication of EO-loaded NPs with antimicrobial properties to resolve the limitations associated with EOs. Nanoparticles protect EOs from heat and UV degradation, ensuring higher stability, flavor retention, and function, thus extending the shelf life of the finished product. Additionally, NPs offer controlled release of EOs for prolonged therapeutic effects. Essential oil-loaded NPs show synergistic antimicrobial action via the enhancement of the diffusion capabilities of EOs through biological membranes. Figure 2 presents various applications of nanoparticles.

### Improvement of Functional Attributes of EOs

The usage of EOs or their constituents is usually hampered by their volatility and chemical instability upon exposure to air, light, moisture, and heat. Thermal and/or oxidatively labile EOs can be degraded during the processing, transportation, storage, and consumption of products containing them to the extent of becoming ineffective, or even dangerous with the formation of toxic derivatives. Various examples portraying these degradative reactions include the degradation of safrole to carcinogenic metabolites, the oxidation of pinene to harmful oxidised derivatives, the di-epoxidation of limonene to the carcinogenic diepoxylimonene, or the formation of oxygenated derivatives of linalool or caryophyllene causing allergenic and skin sensitization properties. Nanoencapsulation presents a novel method for overcoming the aforementioned drawbacks [98]. Nanoencapsulation, as an effective and efficient method, adds a new dimension to increase the stability of EOs and bioactive components by shielding them from direct exposure to natural ambient conditions [99]. Encapsulation also minimises the volatility and toxicity of EOs, improving their water solubility, bioavailability, and bioefficacy by increasing the surface-to-volume ratio, allowing for regulated and site-specific distribution and deep tissue penetration [99]. Essential oils are an attractive alternative for the formation of packaging films, due to their waterproofing qualities and biological activities, transforming packaging into an active material and increasing its value. An example is cellulose-based active packaging material composed of eugenol derivatized with polycarboxylic acid to package wheat flour and other grain items, which exhibited resistance to water absorption to same extend of traditional paper packaging, with good mechanical strength. It also displayed considerable pesticide and insectifuge capabilities, extending the shelf life of the product without altering its natural flavour, taste, or odour. The active biodegradable films with encapsulated EOs demonstrated no cytotoxicity and allowed the sensory characteristics of the food products to be preserved throughout storage. Encapsulation can retain EOs for longer time periods by interacting with the matrix in a physical or chemical manner. Accordingly, in cosmetics, means of encapsulating EOs in a way that is amenable to release through mechanical impacts have been designed. However, the encapsulation of flavours for food applications necessitates moderate regulated release. Encapsulation also facilitates the incorporation of fats into the various matrices used to make packaging materials, as it lowers unfavourable interactions between the lipid phase and the matrix, which is generally hydrophilic in nature [100].

Nanoparticles such as chitosan nanoparticles loaded with essential oils are proven to be safe and efficacious. Jamil et al. demonstrated the safety of cardamom oil-loaded chitosan NPs with their results showing an absence of hemolysis and necrosis in mammalian cells [101].

## 5. Synthesis of Essential Oil-Loaded Nanoparticles

**Figure 3:**
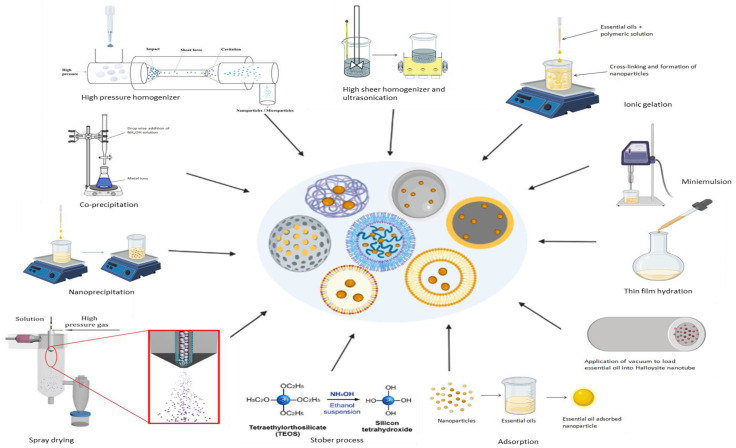
Synthetic methods for nanoparticles.

Various approaches for the manufacturing essential oil-loaded nanoparticles including co-precipitation, high-pressure homogenization, high-speed stirring, ultra-sonication, ionic gelation, mini-emulsion polymerization, nanoprecipitation, spray drying, and the Stöber process have been developed during the last few decades (Figure 3). The behavior of NPs loaded with EOs is greatly influenced by their size, shape, and surface chemistry; therefore, any deviation in the preparation method could result in significant differences in the final product.

### 5.1. Co-Precipitation Method

The co-precipitation method is one of the most widely utilized methods for the synthesis of metal NPs, magnetite NPs, and inorganic NPs owing to its non-toxic nature, cost-effectiveness, and moderate reaction conditions. In this method, the use of metal precipitated in the form of hydroxide from a salt precursor, chlorides, perchlorates, sulphates, and nitrates produced with the help of a base, and sodium, potassium, or ammonium hydroxide in a solvent, results in the production of nanoparticles by means of nucleation and grain formation. The size, shape, and magnetic characteristics of metal and inorganic NPs are influenced by the types and quantities of salts, in addition to the temperature, pH, ionic strength, and mixing rate [102]. Bioactive coating comprising patchouli oil-loaded magnetite NPs was prepared via the co-precipitation of iron precursor in an alkaline solution of patchouli oil. Peppermint oil-, lavender oil-, and basil oil-loaded hydroxyapatite nanoparticles, and mentha oil-, patchouli oil-, vanilla oil-, ylang-ylang oil-, cinnamon oil-, black cumin oil-, nutmeg oil-, clove oil-, rosemary oil-, eugenol-, and limonene-encapsulated magnetite nanoparticles, were prepared using the co-precipitation method.

### 5.2. High-Pressure Homogenization Method

High-pressure homogenization (HPH) under hot and cold conditions is widely used for the preparation of solid lipid nanoparticles (SLN) and nanostructured lipid carriers (NLC). In this approach, under high pressure, a reduction in the size of droplets and particles occurs.

(a) Hot HPH method—Essential oils and melted lipids are combined at a temperature that is 5–10 °C above the solid lipid melting point and either dissolved or uniformly dispersed in the molten lipids. A pre-emulsion is prepared by combining hot aqueous phase containing surfactants and molten lipid under constant stirring and homogenized with a piston-gap homogenizer. SLNs and NLCs are obtained after 3–5 homogenization cycles at a pressure of 500–1500 bars. After homogenization, the nanoemulsions are cooled, causing lipid crystallization and the formation of SLNs and NLCs. This approach was used to fabricate NLCs encapsulated with *Punica granatum* seed oil and menthol with particle sizes of 102.10–115.6 nm and a PDI of 0.2.

(b) Cold HPH method—Essential oils are dissolved/dispersed in molten lipids and rapidly cooled using liquid nitrogen or dry ice. Lipid-EOs combinations are subjected to size reduction to a particle size range of 50–100 µm in a ball mill or mortar to obtain lipid microparticles, which are further suspended in cold aqueous solutions containing surfactants and homogenized at 500 bar pressure at 0–4 °C for 5–10 cycles to obtain SLNs and NLCs [103]. *Melaleuca alternifolia* oil-loaded nanoparticles with particle sizes below 300 nm and a PDI of 0.25 were synthesized using this approach.

### 5.3. High-Speed Stirring and Ultra-Sonication Methods

High-speed stirring (high-shear homogenization) and ultrasonication is associated with ease of handling and a decrease in the use of organic solvents. In this method, EOs and melted lipids are combined at a temperature above the solid lipid melting point. A hot pre-emulsion is prepared by mixing hot aqueous phase-containing surfactants and molten lipid at same temperature with continuous stirring. The prepared emulsion is ultrasonicated using a probe sonicator over nine cycles, with 30 s of sonication separated by 15 s intervals. The final formulations are cooled to room temperature to obtain SLNs and NLCs [103,104]. *Eugenia caryophyllata* and *Melaleuca alternifolia* essential oil-loaded SLN were prepared using high shear homogenization for 5 min at 20,500 rev/min, followed by ultrasonication for ~6 min, and demonstrated particle sizes of 300–700 nm with a PDI of 0.21–0.68.

### 5.4. Ionic Gelation Method

Ionic gelation is based on the ability of polyelectrolytes to crosslink in the presence of counter ions [105]. Essential oils and a polymer are dissolved in a mild acidic medium or water according to their solubility and the resulting solution is slowly added to the solution containing counter ions and stabilizer. Gelation and precipitation, resulting from the complexation of oppositely charged species, lead to spherical-shaped particles. The particle size is reduced to the nanometric range by sonicating the resultant solution and freeze drying immediately at −30 °C for 1 h [106,107]. The ionic gelation process is commonly used to develop essential oils of *Satureja hortensis*, *Zataria multiflora*, *Eugenia caryophyllata*, *Psidium guajava* leaves, and *Nigella Sativa-*, *Homalomena pineodora-*, *Bunium persicum-, Carum copticum-, Satureja khuzistanica-, Thymus capitatus-, Oreganum vulgare-,* and *Citrus reticulate*-loaded chitosan nanoparticles with particle sizes below 300 nm. Lemongrass oil-, chamomile oil-, and tea tree oil-loaded nanocapsules were fabricated using the ionic gelation method.

### 5.5. Miniemulsion Polymerization Method

Miniemulsion polymerization techniques are used for the synthesis of cross-linked polymeric nanoparticles by the addition of an appropriate surfactant and co-stabilizer. During this process, aqueous dispersions of tightly packed, intently distributed small monomeric droplets stabilized against Ostwald ripening and collisional disintegration are produced. The size of the particles formed by miniemulsion polymerization depends on reaction parameters such as the sonication time as well as the concentrations of the initiator, co-stabilizer, and surfactant. High shear mixing results in monomer droplets in a size range 50 to 500 nm, serving as discrete nanoreactors for the formation of polymer nanoparticles. Mass mobility between monomer droplets is prevented in this method; therefore, components can be added to the organic phase prior to shear mixing. Thymol-/carvacrol-loaded polythioether NPs having loading capacity of ≈50% *w*/*w* and encapsulation efficiency of greater than 95% were synthesized using the miniemulsion polymerization method [108]. D-Limonene-loaded methyl methacrylate- and triethylene glycol dimethacrylate co-polymer-based nanoparticles were synthesized using this method.

### 5.6. Nanoprecipitation Method

Nanoprecipitation is the method wherein the precipitation and eventual solidification of polymers are induced via the coating of the polymer at its interface after the displacement of water-miscible semi-polar solvents from a lipophilic solution, resulting in the formation of particles. The nanoprecipitation approach uses two miscible phases: an organic phase and an aqueous phase acting as a solvent (polymer and EO) and a non-solvent, respectively. Essential oil and polymer are dissolved in the organic solvent before being mixed into an aqueous phase containing stabilizer. This results in decreased interfacial tension between the aqueous and organic phases, allowing the organic solvent to diffuse easily into the aqueous phase. Small and narrowly distributed droplets of NPs are instantly formed at the interface of the organic solvent and the aqueous phase during the solvent flow, diffusion, and surface tension. The resulting nanosuspension is freeze dried with 5% mannitol as a cryoprotectant to yield a fine powder of nanoparticles. Encapsulation of essential oil using the nanoprecipitation process is associated with advantages such as reduced process time, simplicity, good reproducibility, scalability, and the formation of submicron nanoparticles with narrow size distributions and high encapsulation efficiencies [98,109]. This approach was used to fabricate starch nanoparticles of bergamot and sweet orange essential oil with particle sizes below 150 nm, a PDI of ~0.2, and an encapsulation efficiency above 80%. Lemongrass-, thymol-, and menthone-loaded polymeric nanoparticles were fabricated using the nanoprecipitation method.

### 5.7. Spray Drying Technique

Spray drying is a commonly used fabrication process for obtaining powder from liquid phase. Spray dryers with vibrating mesh technology use rotary atomizers and pressure nozzles to generate fine droplets. In this method, polymer solutions are introduced by utilizing a peristaltic pump to form polymeric complexes. Separately, an emulsion of oil and surfactant is produced and gradually introduced into the polymer complexes at varied polymer matrix: oil ratios. The various parameters affecting the product in this process include the input temperature, outlet temperature, pump feed flow, and air flow [110]. *Lippia sidoides* oil was incorporated into chitosan and cashew gum nanogels by means of a spray drying technique with particle sizes ranging between 300 and 900 nm and a PDI of 0.3–0.6. Other essential oils incorporated into nanogel using this method were rosemary oil, clove oil, cinnamon oil, and lemongrass oil.

### 5.8. Stöber Process

The Stöber technique is a chemical process used to fabricate silica nanoparticles. The Stöber approach involves the hydrolysis and condensation of a combination of alkoxysilanes such as tetraethyl orthosilicate in a mild basic aqueous medium containing mixtures of alcoholic solvents, water, and ammonia resulting in the development of a silica nanoparticles. The sol–gel process is a modified version of Stöber’s method that is used to fabricate mesoporous silica nanoparticles (MSNs). It involves the hydrolysis and condensation of the alkoxide monomers into a colloidal solution that acts as a precursor to form an ordered gel-like network of polymer or discrete particles, in the presence of an acid or a base catalyst. Another modification of this process involves use of a cationic surfactant in the reaction mixture resulting in spherical particle of submicrometer size monodisperse particles [111]. Silica nanoparticles loaded with essential oils of chamomile, *Artemisia annua*, lemongrass and clove, peppermint, *Pistacia atlantica* were formulated by this method with particle sizes ranging from 20 nm to 500 nm.

### 5.9. Thin Film Hydration, Adsorption and Vacuum Pulling Methods

Thin film hydration method involves the formation of a thin lipid film in a round-bottom flask by the evaporation of organic solvent. Lipids and essential oil are dissolved in absolute ethanol in a round bottom flask. The resultant mixture is desolvated under reduced pressure using a rotary evaporator to obtain a thin film. Subsequently, the thin film is hydrated using water. The thin film hydration method was used for the preparation of liposomes loaded with essential oils such as oregano, artemisia, laurel and eugenol. In the adsorption method, essential oils are adsorbed onto nanoparticles. The adsorption method was used to prepare rosemary oil-loaded TiO_2_ nanoparticles, carvacrol-, eugenol- and fennel oil-loaded ZnO nanoparticles and linalool-, *Thymus vulgaris*-, *Nigella sativa* EO-loaded metal nanoparticles. In the vacuum pulling method, EO is mixed with Halloysite nanotubes and sonicated. The mixture was vacuum filtered and maintained in a vacuum for 30 min to extract air from the inner surfaces of HNTs. Essential oil was loaded into the inner space of HNTs by capillary action until the atmospheric pressure was reached, followed by centrifugation and filtration. Vacuum pulling was used to fabricate thyme-loaded halloysite nanotubes.

Nine different methods for the synthesis of essential oil-loaded nanoparticles, which could successfully synthesize nanoparticles of the desired shape, size, surface chemistry, and physical stability, are reported in the literature. High-pressure homogenization is the most commonly used method for the preparation of SLNs and NLCs due to reasons such as short production periods, ease of manufacturing, organic solvent-free operation, and scale-up feasibility. Essential oil-loaded SLNs and NLCs are usually prepared using the hot homogenization method; however, this method has disadvantages such as product degradation, essential oil loss from the aqueous phase, and unpredictable lipid transitions that can be overcome by means of the cold homogenization method [103]. The ionic gelation method is widely used for the synthesis of polymeric nanoparticles due to its ease of preparation and modification, and lesser requirements of toxic solvents [112]. The co-precipitation method is extensively used for the preparation of EO-loaded magnetite/metal nanoparticles and it is a simpler, faster, and more cost-effective and easily scalable process [113]. Silica nanoparticles loaded with EOs are prepared mostly using the Stöber technique as it yields monodisperse, ordered, nanosized silica particles and can easily be modified [114].

## 6. Nanoparticles as Carriers of EO

Nanoparticles are classified into several groups, depending on their morphology, size, and chemical characteristics. The types of NPs loaded with EOs for antimicrobial effect are discussed below.

### 6.1. Inorganic Nanoparticles

Inorganic nanoparticles are made up of inorganic materials such as hydroxyapatite (Hap), titanium dioxide, and zinc oxide, and are hydrophilic, non-toxic, biocompatible, and highly stable compared to organic materials. Inorganic NPs can be designed with a wide variety of sizes, structures, and geometries. Due to hydroxyapatite, (Ca_5_[PO_4_]_3_OH)_2_ being similar in nature to human hard tissue and having advantages such as nontoxicity, biocompatibility, and osteoconductivity, hydroxyapatite nanoparticles are commonly used as implant materials and drug delivery carriers. Hydroxyapatite activates and modulates regeneration of bone tissue due to its bioactivity and surface chemistry. Therefore, combining essential oils with hydroxyapatite offers an excellent antibacterial strategy to handle trauma or bone fracture-related infections [115]. TiO_2_ and ZnO nanoparticles have been combined with EOs for improvement in packaging properties of materials. TiO_2_ nanoparticles have displayed significant antibacterial activity as a single agent or in combination therapy with essential oils. TiO_2_ scavenges the oxygen and water due to photocatalyst activity therefore can be used in packing materials. Incorporating TiO_2_ into biopolymer-based packaging materials increases their tensile strength, heat resistance, permeability, UV protection and antimicrobial activity [116]. ZnO nanoparticles (ZnO-NPs) are reported to have thermal stability, low toxicity, UV filtration property, and potent antimicrobial activity. It was previously demonstrated that the combination of ZnO-NPs with essential oils exhibits synergistic antimicrobial activity [117].

Badea et al. synthesized peppermint oil-loaded Hap nanoparticles (Hap-P) wherein Hap nanoparticles were formed by means of the co-precipitation method followed by the adsorption of peppermint oil, resulting in ellipsoidal shaped particles with sizes of 19.56 ± 3 nm (Hap) and 21.10 ± 5 nm (Hap-P). The results of the antimicrobial study demonstrated that Hap nanoparticles did not inhibit the growth of microorganisms. The MBC values of Hap-P against the microorganisms ranged between 15.62 and 125 µL/mL and Hap exhibited MIC values over 250 µL/mL. MIC values of Hap-P against *P. aeruginosa, C. parapsilosis, E. faecium, E. coli*, *S. aureus* and MRSA was fond to be 31.25 µL/mL, 31.25 µL/mL, 125 µL/mL, 250 µL/mL, 250 µL/mL and 250 µL/mL, respectively. Thus, the study proved that adsorption of peppermint EO at the surface of Hap nanoparticles could be beneficial for manufacturing of implants that would reduce the post operative infections [115]. Predoi et al. formulated spherical-shaped hydroxyapatite nanoparticles containing essential oils of lavender (HapL) and basil (HapB) with particle sizes of 76.8 ± 5 nm and 63.3 ± 6 nm, respectively, by means of the co-precipitation method followed by the adsorption of Eos, and evaluated their antibacterial efficacy against gram-positive microorganisms, namely MRSA and *S. aureus*, and gram-negative microorganisms such as *E. coli*. Only treatment with Hap was devoid of significant antibacterial activity. However, HapL exhibited significantly enhanced antimicrobial action against all the screened organisms, with MIC values in the range of 0.15 mg/mL to 0.62 mg/mL, in comparison to HapB, which had MIC values of greater than 5 mg/mL. The incorporation of EOs into the Hap microporous structure caused an augmentation of their membrane depolarization effect, resulting in enhancement of antibacterial activity. Therefore, the study revealed utility of Hap nanoparticles as delivery systems for EOs with antibacterial properties in bone reconstruction to reduce infection associated with implants [118]. Alizadeh-Sani et al. fabricated cellulose nanofiber- or whey protein matrix-based packaging materials consisting of TiO_2_ particles and rosemary oil droplets as active components, by means of a casting method, and investigated their antibacterial activity against resistant foodborne pathogenic microorganisms such as *L. monocytogenes* and *S. aureus* that are present in meat. The antimicrobial potential of the packaging material was accredited to active constituents of rosemary oil and formation of reactive oxygen species by TiO_2_. The psychrotrophic bacteria count (PBC) values of lamb meat packaged in the composite films, stored for 15 days at 4 °C, were compared to those of the control samples. The results indicated that the untreated meat sample displayed an initial PBC value of 3.3 log CFU/g and, after storage of 9 days, this value changed to 7.1 log CFU/g. The PBC values of the treated sample were substantially lower than 4.15 log CFU/g for the same storage period and remained below the microbiologically allowed limit (5.3 log CFU/g) for 15 days. Therefore composite films, which caused a decreased rate of PBC growth on raw lamb meat during storage, proved their efficacy as active packaging to extend the shelf life of fresh meat products [116]. Mizielińska et al. developed two types of external coatings using Polyethylene films made of methyl hydroxyl propyl cellulose (MHPC) along with ZnO nanoparticles and carvacrol (ZC1) or geraniol (ZG1) and evaluated for antibacterial activity and for antiviral activity against viruses such as SARS-CoV-2. The findings revealed that MHPC coatings formed by adding ZnO nanoparticles to the carrier containing low levels (0.0125 g) of geraniol or (0.0125 g) of carvacrol (ZG1, ZC1) were active against *S. aureus* (~1 × 10^0^ log CFU/mL)*, E. coli* (~1.54 × 10^4^ log CFU/mL, ~1.00 × 10^4^ log CFU/mL) and *P. syringae* (2.22 × 10^2^ log CFU/mL, 2.78 × 10^2^ log CFU/mL). The incubation of the phages with ZG1 and ZC1 coatings demonstrated reduction in bacteriophage titer by one log. The results suggested that ZG1 and ZC1 coatings showed antiviral activity, as a result of the reduction in the phage titer in the initial phase of incubation was devoid of complete elimination of any active phage particles [117]. Babapour et al. synthesized bionanocomposite films comprising potato starch and ZnO-NP and fennel essential oil (FEO) by means of the casting method. Antimicrobial studies revealed that antimicrobial activity against *S. aureus*, *E. coli*, and *A. flavus* increased with increase in the concentration of ZnO-NP and FEO in films. Films with highest concentrations of ZnO-NP (5%) and FEO (3%) displayed zone of inhibition of 146.15 mm^2^, 124.37 mm^2^, 104.88 mm^2^ against *S. aureus*, *E. coli*, and *A. flavus*, respectively. Therefore, the biocomposite potato starch films demonstrated excellent synergistic effect of ZnO- NP and FEO [119].

### 6.2. Lipid Nanoparticles

Lipid-based NPs are most specifically spherical particles consisting of a lipid bilayer surrounding an inner aqueous compartment. Two major classes of lipid nanoparticles are solid lipid nanoparticles and nanostructure lipid carriers. The various advantages of lipid nanoparticles as delivery vehicles include ease of formulation, biocompatibility, self assembly, high bioavailability, larger pay load transfer capacity, and ease of physico-chemical properties’ modulation to impact their biological activity. The most commonly used lipids used for formulating EO-loaded lipid nanoparticles include glyceryl monostearate (GMS), precirol, stearic acid (SA) and cetyl palmitate. Encapsulation of EOs in SLNs and NLCs could be a unique strategy to overcome the issues associated with EOs such as toxicity, poor stability and pharmacokinetic properties [120].

Bazzaz et al. developed spherical-shaped solid lipid nanoparticles containing *Eugenia caryophyllata* essential oil by means of a high-shear homogenization and ultrasonic method using three different lipids such as glyceryl monostearate, precirol, and stearic acid, along with Tween 80 and poloxamer as surface stabilizers. Three optimized formulations chosen for antimicrobial screening, SLN-SA-EO (F1), SLN-P-GMS-EO (F2), and SLN-GMS-EO (F3), which exhibited particle sizes of 397 ± 10.1 nm, 786 ± 9.11 nm, and 506 ± 4.22 nm, PDI values of 0.215 ± 0.01, 0.48 ± 0.03, and 0.680 ± 0.02, zeta potentials of −20.9 ± 0.3 mV, −21.7 ± 0.5 mV, and −21.7 ± 0.2 mV, and an entrapment efficiency of 70%. The encapsulation of EOs in SLNs caused 2–20-fold reduction in antimicrobial activity as revealed by their MIC values. The MIC values of F1, F2, F3, and EO against *S. typhi* were 0.005 µg/mL, 0.01 µg/mL, 0.01 µg/mL, and 0.1 µg/mL, those against *P. aeruginosa* were 0.01 µg/mL, 0.1 µg/mL, 0.1 µg/mL, and 0.5 µg/mL, those against *S. aureus* were 0.25 µg/mL, 0.5 µg/mL, 0.5 µg/mL, and 0.5 µg/mL, and those against *C. albicans* were 0.1 µg/mL, 0.25 µg/mL, 0.25 µg/mL, and 0.25 µg/mL, respectively [104]. Fathi et al. fabricated NLCs encapsulated with *Punica granatum* seed oil (PGS-NLCs), of 102.10 nm particle size and with a narrow size distribution (PDI = 0.26), using a hot melt homogenization process and investigated their antibacterial efficacy against *S. epidermidis*. An antibacterial assay revealed that PGS-NLCs exhibited stronger antimicrobial activity than *P. granatum* seed oil emulsion. The PGS-NLCs developed in the study were proposed to be used in dentistry and skin-related materials [62]. De Souza et al. developed dental biofilm containing spherical-shaped *Melaleuca alternifolia* essential oil nanoparticles (NPTTO) by means of a high-pressure homogenization method and evaluated antibiofilm activity in human dental biofilm with the use of an in situ model. The particle size, PDI, zeta potential and pH of synthesized nanoparticles were found to be 197.9 nm, 0.242 ± 0.005, 7.12 ± 0.27 mV and 6.4 ± 0.2, respectively. Antimicrobial analysis of the solutions used to test the device in situ model revealed reductions in the colony forming units (CFU) of 34.2%, 51.4%, and 25.8% from 0.12% chlorhexidine solution, 0.3% *M. alternifolia* oil solution, and NPTTO, respectively. Thus, the study substantiated antibiofilm activity of NPTTO through inhibition of commonly occurring microorganisms such as *Candida* species and *Pseudomonas aeruginosa* [121]. Piran et al. fabricated spherically shaped menthol-loaded nanostructured lipid carriers with particle size, PDI and entrapment efficiency of 115.6 nm, 0.2, 98.73% respectively using hot melt homogenization. Evaluation of antimicrobial activity revealed higher activity for NLC in comparison to menthol emulsion. MIC values of menthol emulsion and NLC against *Bacillus cereus* (1000 µg/mL, 125 µg/mL), *Staphylococcus aureus* (1000 µg/mL, 250 µg/mL), *Escherichia coli* (2000 µg/mL, 500 µg/mL), and *Candida albicans* (156 µg/mL, 78 µg/mL). Thus the study substantiated enhanced antibacterial activity of NLC for use in food preservation [122]. Comin et al. fabricated TTO-loaded solid lipid nanoparticles to investigate the activity against *Pseudomonas aeruginosa* adhesion in buccal epithelial cells. In physicochemical evaluation, average size, pH, mean diameter, PDI, and zeta potential were found to be 166 ± 29 nm, 6.3 ± 0.3, 150.2 ± 2 nm, 0.213 ± 0.017, and 8.69 ± 0.80 mV, respectively. The effect of TTO nanoparticles on virulence characteristics such as motility and adherence of *P. aeruginosa* in buccal epithelial cells was confirmed in the study. Both oil and nanoparticles inhibited *P. aeruginosa* mobility and reduced microbe adhesion to buccal cells and biofilm. TTO nanostructure has been shown to be a viable alternative to bacteria that produce biofilms [123]. Saporito et al. fabricated lipid nanoparticles loaded with Eucalyptus essential oil (EO-NLCs) using a high-shear homogenization and ultrasound method as a medical device to improve skin wound healing. Natural lipids such as cocoa butter as a solid lipid and olive oil or sesame oil as a liquid lipid were used. Lecithin was employed as a surfactant to stabilise nanoparticles and avoid aggregation. The nanoparticles were round-shaped and their particle size, PDI, and zeta potential were 220–300 nm, 0.5, and −22.07 mV, respectively. The eucalyptus oil, in free and encapsulated form, exhibited an MIC value of 3 mg/mL against *Staphylococcus aureus*. However, improved antibacterial action against *Streptococcus pyogenes* was observed for EO-NLCs with MIC values decreasing from 1.5 mg/mL of that of free oil to 0.75 mg/mL for EO-NLs [124].

### 6.3. Liposomes

Liposomes are phospholipid vesicles with separate aqueous compartments and are comprised of one or more concentric lipid bilayers. Their unique ability to entrap lipophilic molecules in the lipid bilayer and hydrophilic molecules in the aqueous core allows them to encapsulate a diverse range of therapeutic candidates. Various advantages of liposome include ease of formulation, high bioavailability, biocompatibility, self assembly, larger pay load transfer capacity, and ease of physico-chemical properties’ modulation to impact their biological activity. Incorporation of EOs into nanoliposomes is a promising technique for decreasing toxicity, preventing degradation, and increasing the bioavailability of EO [125]. Ethosomes are modified liposomes made up of phospholipids with high concentrations of ethanol and water. Ethanol assists ethosomes to pass through pores and deeply penetrate into deeper skin layers, making them more efficient than traditional transdermal nanocarriers [126,127].

Aguilar-Perez et al. prepared oregano essential oil-entrapped small unilamellar vesicle nanoliposomes using the thin film hydration-sonication method. The particle size range, PDI, zeta potential, and entrapment efficiency of the developed nanoliposomes were found to be 77.46 ± 0.66 nm to 110.4 ± 0.98 nm, 0.413 ± 0.015, 36.94 ± 0.36 mV, and 79.55 ± 6.9%, respectively. In vitro antifungal evaluation at a concentration of 1.5 µL/mL demonstrated the highest mycelial growth inhibition (81.66 ± 0.86%) for nanoliposomes against *Trichophyton rubrum* in comparison to the other formulations. Thus, their study proved the potential of oregano EO nanoliposome in antifungal treatment [128]. Sinico et al. developed a positively charged multi lamellar (MLV) and unilamellar (SUV) liposomal formulation loaded with *Artemisia arborescens* L. essential oil by means of film and sonication methods. The resultant formulations displayed particle sizes and entrapment efficiencies in the range 70–150 nm and 60–74%, respectively. Antiviral activity is expressed in terms of the viral cytopathic effect (CPE). The entrapment of EO in SUV and MLV liposomal formulation resulted in greater antiviral activity substantiated by a reduction in %CPE. The CPE values of free EO (100 µg/mL), P90H (hydrogenated soy phosphatidylcholine) SUV (100 µg/mL), P90 (non-hydrogenated soy phosphatidylcholine) SUV (100 µg/mL), P90 MLV (100 mg/mL), and P90H MLV (50 mg/mL) were found to be 22.86%, 21.1%, 8.1%, 100%, and 100%, respectively. The EC_50_ values of P90H MLV and P90 MLV were observed to be 18.5 and 43.6 mg/mL, respectively. Essential oil, P90H SUV and P90 SUV exhibited EC_50_ values above 100 µg/mL. Thus, the antiherpetic activity of MLV was found to be higher as compared to SUV against HSV-1. The developed liposomes could be beneficial in enhancing the delivery of antiviral EOs to target cells [129]. Wu et al. fabricated nanoliposome composite containing Laurel essential oil (LEO) and AgNPs (Lip-LEO-AgNPs) using a film hydration approach for controlled release. The particle size, PDI and zeta potential were found to be 200 nm, 0.27 and −26 mV, respectively. Lip-LEO-AgNPs was mixed with chitosan to form polyethylene (PE-CS) for pork packaging. Antibacterial evaluation of the film coating against *S. aureus* and *E. coli* that were responsible for the decomposition and putrefaction of meat revealed that PE and PE-CS films were devoid of antibacterial activity, and PC-Lip/LEO/AgNPs exhibited the highest antibacterial activity (the zone of inhibition was ~5 mm for *S. aureus* and ~1 mm for *E. coli*). Thus, the outcomes of the study substantiated synergism in developed films for their use in packaging of functional foods for extended shelf-life [130]. Jin et al. fabricated eugenol entrapped ethosome nanoparticles (ELG-NPs) with a particle size, zeta potential, and entrapment efficiency of 44.21 nm, −40.3 ± 1.7 mV, and 82%, respectively, and investigated their antibacterial efficiency against fruit anthracnose post-harvest for 6 days. Anthracnose is caused by pathogens that are infected at any stage from preharvest treatment to postharvest storage and transportation. The results showed that mycelia growth inhibition caused by eugenol against *Colletotrichum musae, Colletotrichum fragariae, Colletotrichum gloeosporioides, and Colletotrichum gloeosporioides* were only 83.71%, 82.90%, 86.89%, and 83.72%, respectively, whereas the inhibition rates for the ELG-NPs were significantly higher at 95.23%, 90.08%, 89.43%, and 94.19%, respectively. The ELG-NPs exhibited an antibacterial activity (>93%) against fruit pathogens which was greater than that of eugenol and showed 100% inhibition of the anthracnose incidence in post-harvest loquat after 6 days. Eugenol and ELG-NPs could completely inhibit the growth of bacteria at concentrations higher than 87 µL/L. The nanoencapsulated eugenol showed a sustained release profile for a longer duration, and thereby caused more effective inhibition of the growth of pathogenic bacteria than eugenol [131].

### 6.4. Magnetite Nanoparticles

Magnetic iron oxide nanoparticles are researched intensively for a variety of applications such as drug delivery, magnetic resonance imaging, and theranostics. Their utility stems from the possibility of utilizing magnetic fields to drive nanoparticles to the desired location, resulting in drug heating and controlled release. Magnetic nanoparticles are one of the promising choices for the development of EO-based antimicrobial nanosystems due to their biocompatibility, biodegradability, lack of toxicity and targetability.

Anghel et al. developed magnetite NP containing *Mentha piperita* EO with particle size 5 nm by means of the co-precipitation method. Nanosystem was coated on the surface of catheter device to evaluate its anti-adherence and antibiofilm activity. The result demonstrated that the biofilm developed on the coated catheter device decreased significantly as compared to the uncoated surface. The nanosystem-coated catheter device exhibited a viable cell count of ~4 log CFU/mL, ~4.5 log CFU/mL, and ~5 log CFU/mL at 24 h, 48 h, and 72 h, respectively, while the uncoated device had a count of ~6 log CFU/mL, ~8 log CFU/mL, and ~10 log CFU/mL at 24 h, 48 h, and 72 h, respectively [132]. Rădulescu et al. fabricated a biocompatible wound dressing composed of MNPs and patchouli EO solutions by means of the co-precipitation method. The developed MNPs exhibited a particle size of 7.5 nm. The results demonstrated significant antibacterial activity against *Staphylococcus aureus* biofilms with a two-fold reduction in viable cells embedded in biofilms after 24 h and 48 h incubation with bioactive coatings. The effect of coating lasted up to 72 h. Thus, the developed bioactive MNP coated wound dressing could be utilized for the efficient management of wound infections [133]. Bilcu et al. developed MNPs coated with patchouli (PAT), vanilla (VAN), and ylang-ylang (YLG) EOs with particle sizes below 20 nm by means of the co-precipitation method. The results of their comparative antibacterial study showed a significant reduction in the growth of *S. aureus* and *K. pneumoniae*. The viable *S. aureus* cell counts of the PAT-MNP-, VAN-MNP-, YLG-MNP-, and MNP-coated catheters and the uncoated catheter were observed to be ~1 × 10^3^ log CFU/mL, ~1 × 10^4^ log CFU/mL, ~1 × 10^3^ log CFU/mL, ~1 × 10^3^ log CFU/mL, and ~1 × 10^4^ log CFU/mL, respectively, at 72 h, while for *K. pneumoniae*, these values were observed to be ~1 × 10^6^ log CFU/mL, ~1 × 10^6^ log CFU/mL, ~1 × 10^5^ log CFU/mL, ~1 × 10^6^ log CFU/mL, and ~1 × 10^5^ log CFU/mL, respectively [134]. Anghel et al. fabricated MNPs functionalized with *Cinnamomum verum* EO (MNP-CV) with a particle size 9.4 nm, and these were transferred by means of a matrix-assisted pulsed laser evaporation technique to form a homogeneous thin coating on the surfaces of gastrostomy tubes (G-tubes). The result suggested that the biofilm growth was significantly decreased by the application of the MNP-CV coating on the surface. The rate of inhibition of *S. aureus* colonization ranged from more than four-fold for incipient biofilms to three-fold for mature biofilms, and the rate of *E. coli* biofilm inhibition ranged from 2.5-fold for inceptive biofilms to 2-fold for mature biofilms. MNP-CV films could be utilised for the antibacterial protection of medical surfaces and devices for patients suffering from debilitating conditions [135]. Negut et al. developed *Nigella sativa* EO-functionalized MNPs by means of a modified co-precipitation method. The developed nanoparticles were combined with a polymeric matrix of poly (lactic-co-glycolic acid) and deposited on glass and silicone surfaces using the MAPLE process. Antimicrobial evaluation demonstrated that the MIC values of MNP-NS against *E. coli, S. aureus* and *C. albicans* were 1.25 mg/mL, 0.62 mg/mL and 0.62 mg/mL, respectively. The antimicrobial effect of MNP-NS and PLGA-MNP-NS were evaluated against biofilm formed by *S. aureus* (~1 × 10^8^ log CFU/mL, ~1 × 10^7^ log CFU/mL), *E. coli* (~1 × 10^8^ log CFU/mL, ~1 × 10^9^ log CFU/mL) and *C. albicans* (~1 × 10^4^ log CFU/mL, ~1 × 10^5^ log CFU/mL) [136]. Mousavi et al. fabricated Fe_3_O_4_-MgO nanoparticles using nutmeg EO by chemical and green synthesis. The nanoparticles developed exhibited particle size of 10–15 nm. The result of antimicrobial activity demonstrated that green synthesized nanoparticles showed greater rate of inhibition. MIC values of nutmeg EO, chemically synthesized Fe_3_O_4_-MgO and green synthesized Fe_3_O_4_-MgO against *E. coli* (125 µg/mL, 62.5 µg/mL, 31.25 µg/mL), *P. aeruginosa* (250 µg/mL, 125 µg/mL, 6.25 µg/mL), *S. aureus* (500 µg/mL, 250 µg/mL, 125 µg/mL) and *E. faecalis* (500 µg/mL, 250 µg/mL, 125 µg/mL) were as shown [137]. Grumezescu et al. developed spherical-shaped eugenol-functionalized MNPs with particle sizes of less than 10 nm using the co-precipitation method, and these were embedded in poly(3-hydroxybutyric acid-co-3-hydroxyvaleric acid)–polyvinyl alcohol microspheres using an oil-in-water emulsion approach. The microspheres were coated on the glass surface by means of the MAPLE technique. The nanocoated surface showed great antiadherent and antibiofilm efficiency against *S. aureus* and *P. aeruginosa* biofilms. The coated surface exhibited viable cell counts of ~1 × 10^4^ log CFU/mL, ~1 × 10^4^ log CFU/mL, and ~1 × 10^5^ log CFU/mL at 24 h, 48 h, and 72 h, respectively, as compared to the uncoated surface, which exhibited values of ~1 × 10^6^ log CFU/mL, ~1 × 10^6^ log CFU/mL, and ~1 × 10^7^ log CFU/mL at 24 h, 48 h, and 72 h, respectively. Thus, the developed nanoparticles demonstrated potential in terms of the inhibition of the adhesion and biofilm formation of microorganisms on the surfaces of medical devices and other materials [138]. Anghel et al. synthesized eugenol- or limonene-functionalized MNPs by means of wet chemical precipitation and the use of coated-on wound dressings. The results revealed that the viable cell count of *S. aureus* and *P. aeruginosa* biofilm significantly decreased in nanocoated dressing as compared to uncoated dressing. Limonene-based and eugenol-based nanocoats affected the incipient biofilm formation and mature biofilm development. The limonene-based nanocoat exhibited a viable cell count of ~6 log CFU/mL, ~5.5 log CFU/mL, and ~5 log CFU/mL at 24 h, 48 h, and 72 h, respectively, while the eugenol-based nanocoat showed values of ~5 log CFU/mL, ~6 log CFU/mL, and ~5 log CFU/mL at 24 h, 48 h, and 72 h, respectively. Thus, application of nanocoated wound dressing could minimizes infection associated with implanted devices [102,139]. Miguel et al. prepared magnetite/oleic acid nanoparticles functionalized with EOs (*Eugenia caryophyllata* or *Rosmarinus officinalis*), generated by means of a microwave precipitation method, which was able to suppress adhesion and fungal biofilm growth on functionalized catheter samples. The adherence of various *Candida* clinical species (*C. albicans, C. tropicalis, C. krusei, and C. glabrata*) to catheter surface devices was strongly reduced by magnetite/oleic acid nanoparticles functionalized with *E. caryophyllata* oil and, as a result, biofilm formation was prevented. The biofilm formation of clinical *C. albicans* and *C. tropicalis* strains on catheter surfaces was similarly effectively suppressed by *R. officinalis* essential oil-coated nanoparticles, as measured by confocal laser scanning microscopy and viable cell counts [2]. Anghel et al. fabricated MNP functionalized with *Anethum graveolens* (AG) and *Salvia officinalis* (SO) EOs of 15 ± 2 nm particle size by means of wet chemical precipitation, coated rayon/polyester wound dressing (WD) surface resistant to *Candida albicans* adhesion, colonization, and biofilm formation. Viable cell counts assay revealed that both WD@FMNPs-AG and WD@FMNPs-SO have a significant anti-biofilm potential, as demonstrated by the significant decrease in *C. albicans* biofilm embedded viable cell counts, recovered from the fungal biofilms of 48 and 72 h. WD@FMNPs-AG exhibited viable cell count of ~10^3^ log CFU/mL, ~10^4^ log CFU/mL and ~10^3^ log CFU/mL at 24 h, 48 h, and 72 h, respectively, while WD@FMNPs-SO had a count of ~10^3^ log CFU/mL, ~10^3^ log CFU/mL, and ~10^4^ log CFU/mL at 24 h, 48 h, and 72 h, respectively, as compared to unmodified WD and WD@FMNPs. The obtained results revealed that the nanobiocoatings preferentially inhibit the early stages of biofilm formation (after 24 h), but also reduce the formation and development of mature biofilms [140].

### 6.5. Metal Nanoparticles

Metal NPs have size-dependent characteristics and catalytic activity that are regulated by respective metal ion. Metals such as gold and silver in the form of oxide or salt have been widely employed as antimicrobial agents in agriculture and healthcare industry for ages due to their biocidal action. Recently, their applications have expanded to metal surfaces and coatings, chelates, and nanomaterials, due to recent advances in material science. Particularly, capped metal NPs have a substantially better interaction with viruses and host cells than bare metal NPs [141,142]. Toxicity and resistance attributed to metal NPs and essential oils limit their use. Therefore, to overcome this problem EOs can be loaded onto metal NPs to achieve synergism in antimicrobial activity.

Jabir et al. synthesized glutathione-modified gold nanoparticles with or without linalool (LIN-GNPs, GNPs) with particle sizes in the range of 15–20 nm and 5–11 nm, respectively, and evaluated their antibacterial effectiveness against gram-positive bacteria (*Staphylococcus aureus*), gram-negative bacteria (*Escherichia coli*), and a parasite (*Leishmania tropica*). The results revealed that all strains were sensitive to linalool, with MIC values ranging from 4.62 mg/mL to 6 mg/mL. MIC and MBC of linalool for *E. coli* was 0.77 mg/mL and 0.77 mg/mL, and for *S. aureus* was 1.54 mg/mL and 3.08 mg/mL, respectively. Cytotoxic rate of GNPs and LIN-GNPsat a concentration of 10 mg/mL against *L. tropica* was found to be 38.5% and 72.4%, indicating moderate and significant antiparasitic activity, respectively. Thus, the developed LIN-GNPs proved to have higher antimicrobial activity as compared to GNPs [143]. Aldosary et al. synthesized *Thymus vulgaris* EO-loaded silver nanoparticles (TSNPs) with an average size of 8–105 nm and evaluated their antibacterial properties. Results demonstrated zone of inhibition for TSNPs and water, hexane and ethanolic extract of TEO against *Escherichia coli, Klebsiella oxytoca, Klebsiella pneumoniae, Pseudomonas aeruginosa, Acinetobacter baumanii, Enterobacter aerogenes, Streptococcus dgalacticae, Staphylococcus epidermidis, Streptococcus pyogenes, Staphylococcus aureus,* and *Candida albicans* in range of 15–28 mm, 13–24 mm, 15–35 mm and 16–31 mm, respectively. The antimicrobial efficacy of TSNPs against *A. baumanii* and *St. epidermidis* was found to be greater than that of AgNO_3_. Therefore, it was confirmed that thyme essential oil-loaded silver nanoparticles could be used as antimicrobial agents with synergistic effect [69]. Manju et al. developed spherical-shaped gold nanoparticles using *Nigella sativa* essential oil (NsEO-AuNPs) of particle size in the range of 15.6 nm and 28.4 nm. Evaluation of antibacterial effect demonstrated that at a concentration of 10 µg/mL, NsEO-AuNPs displayed better antibacterial activity against gram-positive bacteria (*Staphylococcus aureus*) (16 mm) than gram-negative bacteria (*Vibrio harveyii*) (5 mm). *S aureus* and *V. harveyii* revealed well-developed biofilms under light and confocal laser scanning microscopic examination, whereas treatment with NsEO-AuNPs (20–80 µg/mL) reduced biofilm development in a dose-dependent manner. Both *S. aureus* and *V. harveyii* exhibited fragmented and resistant biofilm architectures at higher concentrations of NsEO-AuNPs (80 µg/mL). Thus, the synthesized nanoparticles could be of greater therapeutic potential in infectious bacteria [144]. Vilas et al. fabricated 12–26 nm sized, highly pure, crystalline silver nanoparticles biosynthesized using essential oil of *Myristica fragrans*. The results obtained using the agar-well diffusion method demonstrated the significant antibacterial activity of the silver colloid against gram-positive *Staphylococcus aureus* (inhibition zone −12 mm) and gram-negative *Escherichia coli* (inhibition zone—14 mm). Muniyappan et al. utilized *Curcuma pseudomontana* EO for the biosynthesis of gold nanoparticles (NGPs). Testing with two gram-positive bacteria (*B. subtilis* and *P. aeruginosa*) and two gram-negative bacteria (*S. aureus* and *E. coli*) revealed the antibacterial activity of EO and NGPs (MIC value of 100 mg/mL). Vidya et al. biosynthesized NSPs using *Coleus aromatics* EO and observed ZOI of 20 and 30 mm against *S. aureus* and *E. coli*, respectively [145,146,147,148].

### 6.6. Nanogels

Nanogels are three-dimensional networks composed of cross-linked polymer chains. Nanogels have qualities of both hydrogel and nanoparticle attributed to their nanometer-sized hydrogel particles. Nanogels are obtained from polymeric precursors or by heterogeneous monomer polymerization. Nanogels are commonly employed to transport physiologically active low or high-molecular-weight molecules or biomacromolecules. Nanogels are suitable for the delivery of various hydrophobic and hydrophilic drugs. Nangels have potential applications in antimicrobial treatment [149].

Mohsenabadi et al. fabricated biodegradable starch-CMC film composed of benzoic acid (BA) and chitosan covalently bonded nanogel, encapsulating rosemary essential oil (REO), using a self-assembly method. Spherically shaped REO-loaded CS–BA nanoparticles had particle sizes of less than 100 nm and an entrapment efficiency of 80%. The water solubility, water vapour permeability, tensile stress, and elongation values of nanogel-based starch-CMC films were in the range of 45.8 ± 1.6–52.7 ± 1.0%, 2.64 ± 0.08–3.04 ± 0.13 gPa^−1^s^−1^m^−1^, 0.325 ± 0.092–1.203 ± 0.315 MPa, and 85.57 ± 45.51–151.44 ± 36.50%, respectively. The MIC values of free REO-, CS–BA nanogel-, and CS–BA nanogel-encapsulated REO against *S. aureus* were observed to be 200 μg/mL, 80 μg/mL, and 40 μg/mL, respectively, suggesting significant antimicrobial activity for nanogel [150]. Barbosa de Carvalho et al. developed cinnamic acid (CA) grafted chitosan (CS) nanogel containing clove and cinnamon oil (CS-CA-Cl and CS-CA-Ci) with average diameter, zeta potential and encapsulation efficiency of 176.0 ± 54.3 nm and 263.0 ± 81.4 nm, −75.98 and −69.74, 74% and 85%, respectively, proposed for treatment of dermatophytosis infection caused by the fungus *Microsporum canis*. Evaluation of the antifungal activity revealed that the MIC values of clove oil and cinnamon oil against *M. canis* was 133 μg/mL. At 266.0 μg/mL concentration (2 times MIC), the two EOs demonstrated highest inhibition, with no significant difference between the results. CS-CA showed no significant activity against *M. Canis* in the concentration range of 0.6 μg/mL to 2400 μg/mL whereas CS-CA-Ci and CS-CA-Cl nanogels exhibited MIC equivalent to 400 μg/mL. Thus, EO-loaded CA-CS exhibited greater antifungal activity against *M. Canis* compared to plain CA-CS substantiating its therapeutic effectiveness in dermatophytosis [151]. Abreu et al. developed spherical-shaped chitosan and cashew gum nanogels loaded with *Lippia sidoides* oil (LSO) by means of the spray drying method with a particle size, PDI, and zeta potential of the nanoparticle in the range of 335 ± 116–899 ± 21 nm, 0.373–0.633, and 4.0 ± 0.8–49.6 ± 1.3 mV, respectively. Nanogels developed with varied concentrations containing LSO were evaluated for larvicidal activity against *St. aegypti* larvae. Nanogel with 5% cashew gum concentration showed highest mortality rates of 55% and 87% after 24 h and 48 h exposure, respectively. However nanogel with 10% cashew gum concentration exhibited 100% mortality rate after 24 h exposure proving its potential larvicidal activity [110]. Almeida et al. produced hydrogel using lemongrass oil (LG)-encapsulated poly(d,l-lactide-co-glycolide) nanoparticles (LGNP) and investigated its in vitro anti-herpetic efficacy. The developed nanoparticles exhibited mean diameter, PDI and zeta potential of 217.1 nm, 0.481 ± 0.023 and 20.5 mV, respectively. The entrapment efficiency of LGNP-, LG-, and LGNP-loaded hydrogel was found to be 70.68% ± 5.2, 63.64% ± 2.8, and 57.01% ± 2.9, respectively. Antiviral activities of the developed formulations were tested against HSV-1 and HSV-2 using a viral titer reduction assay. LGNP-loaded hydrogel efficiently suppressed both viral strains at very low concentration of LG (0.74 µg/mL) with %Inhibition of 74.9%, which was 42.16, 8.76, and 2.23 times lower than that of free oil-, LGNP-, and LG-loaded hydrogel, respectively. The results suggested effectiveness of hydrogels containing volatile oil-loaded nanoparticles, in terms of lowest EC_50_ values of 11.59–1.32 µg/mL and 6.69–1.34 µg/mL against HSV-1 and HSV-2, respectively, in comparison to free oil- and LG-loaded NPs. Thus, the developed nanoparticles proved to be a potential delivery system for topical herpes therapy [152]. Rashidipour et al. fabricated nanogel comprising chitosan-loaded and *Satureja khuzistanica jamzad* (SKJ) essential oil extracts with a particle size, zeta potential, PDI, and encapsulation efficiency of 571.00 nm, 67.2 mV, 0.451, and 30.74% and investigated its antibacterial properties. The MIC of CS, SKJ and SKJ-CS against various organisms ranged from 3.9 to 250 µg/mL, from 7.8 to 250 µg/mL, and from 7.8 to 500 µg/mL, respectively [153].

### 6.7. Polymeric Nanoparticles

Polymeric nanoparticles consist of solid colloidal particles in the size range 10 nm–1 μm. Morphology of these nanoparticles is easily modifiable and several polymers such as alginic acid, polylactide-co-glycolide, Eudragit, starch, polylactic acid, gelatin, chitosan, and polycaprolactone are used in the construction of nanocarriers. The various methods used to synthesize polymeric NPs include emulsification, ionic gelation, and nanoprecipitation. The multiple means of delivering drugs in polymeric NPs include encapsulation in the NP core, entrapment in the polymer matrix, chemical bonding to the polymer, or attachment to the NP surface.

Feyzioglu et al. fabricated chitosan NPs loaded with *Satureja hortensis* EO using an ionic gelation method. The NPs exhibited diameter, zeta potential and encapsulation efficiency in the range of 140.25 nm to 237.60 nm, −7.54 mV to −21.12 mV, and 35.07% to 40.70%, respectively. The results suggested that the antimicrobial activity of the nanoparticles was less than 0.01–11.79 ± 0.33 log_10_ CFU/mL, 0.01–11.72 ± 0.03 log_10_ CFU/mL, and 0.01–13.55 ± 0.05 log_10_ CFU/mL against *E. coli, L. monocytogens*, and *S. aureus*, respectively, and the nanoparticles loaded with 1.5% EO showed the highest antimicrobial activity [67]. Mohammadi et al. fabricated *Zataria multiflora* essential oil encapsulated in chitosan nanoparticles (ZEO-CSNPs), with an average size of 125–175 nm, using the ionic gelation technique and evaluated the antifungal activity of oil against *Botrytis cinerea Pers*., the microorganism causing grey mould disease. The increase in concentration of ZEO decreased the drug encapsulation and loading efficiency of ZEO from 45.24% to 3.26% and from 9.05% to 5.22%, respectively. Chitosan nanoparticles, at 1500 ppm concentration, significantly reduced both the disease intensity and the prevalence of *Botrytis*-inoculated strawberries stored over 7 days at 4 °C followed by 2–3 days at 20 °C. At day 9, 1500 ppm of ZEO-CSNPs exhibited the lowest percentage of infected strawberries (16.67%) in comparison to CSNPs (66.67%). These findings showed that CSNPs can be used for the controlled release of antifungal EOs to improve their activity [154]. Hasheminejad et al. synthesized four distinct coating dispersions containing chitosan-, clove essential oil-, chitosan nanoparticle-, and clove essential oil-loaded chitosan nanoparticles (CEO-CSNPs) with particle size distributions ranging from 40 to 100 nm using an emulsion-ionic gelation approach and examined their effect on the shelf life and quality of barely processed pomegranate arils stored at 5 °C. Evaluation of the microbial activity for 54 days of cold storage revealed that aril coated with CSs, CEOs, CSNPs, and CEO-CSNPs had delayed incidences of fungal decay, whereas uncoated arils turned out to be unsuitable at day 18. The percentage of antifungal index for CS, free CEO, CSNPs, and CEO-CSNPs, synthesized using two mass ratios of CS to TPP of 1:1 and 1.6:1, were determined against *A. niger* at various concentrations (3–0.187 mg/mL). The rate of inhibition of radial mycelial growth by these CEO-CSNPs was concentration dependent. At 1.5 mg/mL, the CEO-CSNPs (1:1) completely inhibited fungal mycelial growth. Mycelial growth dropped significantly in the range of 0.187–1.5 mg/mL for the CSs, CEOs, and CSNPs; however, no significant decrease in mycelial growth was observed in the range of 1.5–3 mg/mL. The determined antifungal activity decreased from CEO-CSNP to CS in the following order: CEO-CSNPs > CSNPs > CEO > CS. During 54 days of cold storage, the CEO-CSNPs dispersion was observed to be the most effective coating for the extension of the microbiological shelf life of ready-to-eat pomegranate arils [155,156]. Zhang et al. developed spherical-shaped chitosan nanoparticles of average size 40–96 nm loaded with essential oil from guava leaves using two step nanoencapsulation processes including oil-in-water emulsification and the ionic gelation method and investigated their antibacterial effect against the multidrug-resistant *K. pneumoniae*. The results revealed that chitosan-loaded essential oil showed 13%, 52%, and 96% inhibition rates at 10 µg/mL, 60 µg/mL, and 100 µg/mL concentrations, revealing 100 µg/mL as the MIC. Thus, EO-loaded CSNPs exhibited potential antibacterial activity [107]. Dawaba et al. developed spherical-shaped chitosan nanoparticles encapsulating essential oil of *Nigella Sativa* with particle sizes ranging from 361 to 1750 nm using the ionic gelation process. The optimized formulation containing chitosan and benzoic acid ratio (2:1) resulted in minimum-sized particles (341 nm) and the highest entrapment efficiency (98%). Optimized nanoparticles (ZOI = 5.5 cm) were observed to be more efficacious than the pure oil (zone of inhibition = 3.6 cm) in an antibacterial activity assay against *S. aureus* [55]. Prado da silva et al. fabricated tea tree oil-loaded chitosan-poly(-caprolactone) core-shell nanocapsules (NC-TTO-CS) for topical acne treatment with average size, PDI, zeta potential, and pH values of 268.0 ± 3.8 nm, 0.204 ± 0.9, +31.0 ± 1.8 mV, and 5.06 ± 0.17, respectively. The results demonstrated that MIC values of TTO, NC-CS, and NC-TTO-CS against *C. acnes* were 0.56% *v*/*v*, greater than 0.56% *v*/*v*, and 0.14% *v*/*v*, respectively. Four-fold enhancement in antibacterial activity observed for NC-TTO-CS against *C. acnes* was attributed to synergism between TTO and CS proving nanocapsules as a promising vehicle for TTO topical delivery in acne therapy [157]. Rozman et al. synthesized *Homalomenapineodora* essential oil-loaded chitosan nanoparticles (HP-CSNPs) using an ion gelation method to suppress diabetic microorganisms. The developed nanoparticles were spherical-shaped and were measured to be 70 nm in diameter with a significant surface charge of +24.10 mV. The MIC values of HPEO, CSNPs, and HP-CSNPs against *Bacillus cereus-, Bacillus subtilis-, Staphylococcus aureus-*, *and methicilin-resistant Staphylococcus aureus, Escherichia coli, Proteus mirabilis, Yersinia sp., Klebsiella pneumoniae, Shigella boydii, Salmonella typhimurium, Acinetobacter anitratus, Pseudomonas aeruginosa, Candida albicans*, and *Candida utilis* were in the range of 156.25–1250 µg/mL, 78–10000 µg/mL, and 4.88–78.00 μg/mL, respectively. HP-CSNPs exhibited greater MIC (78 μg/mL) against *P. aeruginosa* and the lowest MIC (4.88 μg/mL) against *B. cereus, Yersinia sp., E. coli, A. anitratus,* and *S. typhimurium*. The fractional inhibitory concentration index, determined using the checkerboard assay for all test microorganisms, was ≤0.5, indicating a synergism effect between CSNP and HPEO [158]. Esmaeili and Asgari developed spherical-shaped chitosan nanoparticles loaded with *Carum copticum* essential oil (CEO-CSNPs) using TPP and sodium hexametaphosphate as crosslinkers with average sizes of 30–80 nm by means of an emulsion-ionic gelation technique. Results illustrated that the CEO encapsulated in chitosan nanoparticles displayed the highest antibacterial activity as compared to free CEO. Average ZOIs were against *S. aureus* (11.3 mm, 9.7 mm, 6.7 mm), *S. epidermidis* (12.3 mm, 9.3 mm, 9 mm)*, B. cereus* (10.7 mm, 8.8 mm, 7 mm)*, E. coli* (1 mm, 7.8 mm, and 6.7 mm)*, S. typhimurium* (9.5 mm, 6.8 mm, 6.2 mm), and *P. vulgaris* (8 mm, 7.2 mm, and 5.8 mm), for CEO-CSNPs, CEO, and CSNPs, respectively. This indicated that all of the samples exhibited greater ZOI against gram-positive bacteria than gram-negative bacteria [159]. Granata et al. synthesized chitosan nanoparticles loaded with EOs from *Thymus capitatus* (Th-CNPs) and *Origanum vulgare* (Or-CNPs) using ionotropic gelation. The nanoparticles displayed high encapsulation efficiency (80–83%) and zeta potential values of +44 ± 2 mV and +46 ± 2 mV, respectively. Results illustrated that Th-CNPs, displayed the lowest MIC value (0.03 mg/mL), against *L. monocytogenes* and slightly higher MIC values (0.06 and 0.12 mg/mL) against *Staphylococcus aureus* and *Escherichia coli.* Or-CNPs demonstrated lowest MIC values (0.03 mg/mL) against *L. monocytogenes* and *S. Aureus* and highest MIC value (0.06 mg/mL) against *E. coli*. The finding suggested usefulness of developed nanoparticles against prevent of foodborne infections [160]. Song et al. fabricated Mandarin essential oil (MEO)-encapsulated chitosan nanoparticles (CSNPs) with high encapsulation efficiency, particle size and zeta potential as 67.32–82.35%, 131.3–161.9 nm, and >30 mV. The ZOI of MEO-CSNPs with varied chitosan/MEO ratios against *S. aureus* and *E. coli* ranged from 13.13 mm to 18.22 mm and from 12.51 mm to 17.44 mm, respectively. Antibiofilm activity evaluation revealed thick biofilm for untreated group and reduction in the number of biofilm-wrapped bacteria, thickness and biofilm formation following MEO-CSNPs treatment substantiating utility of developed nanoparticles to prevent biofilm formation [161]. Jiang et al. synthesized zein-pectin composite nanoparticles (ZCPs)with an average particle size, PDI, zeta potential of 660.8 ± 8.1 nm, 0.271 ± 0.004, and 31.23 ± 0.70 mV, respectively by antisolvent approach to stabilise cinnamon essential oil pickering emulsions (ZCCPEs) as an antibacterial strategy. The results revealed that inhibition rate of ZCCPEs against *Alternaria alternata* at 48 h and 96 h was in the range of 18.06–100% and 6.255–100% and against *Botrytis cinerea* was in the range of 46.59–100% and −1.91–100% respectively. MICs of ZCCPEs against *Alternaria alternata* and *Botrytis cinerea* were 0.14 µL/mL and 0.08 µL/mL, respectively [23]. Zhang et al. developed thymol-loaded zein nanoparticles (TZNP) stabilised with sodium caseinate (SC) and chitosan hydrochloride (CHC) using liquid–liquid dispersion approach. Effective adsorption of SC onto the zein nanoparticle surface caused the zeta potential of zein nanoparticles to reverse from positive to negative (in the range of −33.60 to −38.95 mV) and an increase in particle size from 176.85 nm to 204.75 nm with a PDI of less than 0.2. Free thymol, SC-TZNPs (0.052 mg/mL) and CHC-SC-TZNPs (0.020 mg/mL) caused complete inhibition of *S. aureus*, but showed no activity against *E. coli*, *P. aeruginosa* and *C. albicans*. CHC-SC-TZNPs with SC to CHC 1:4 mass ratio exhibited the longest and most significant inhibitory effect during the entire testing time [162]. Kang Li et al. fabricated antimicrobial films using sodium caseinate (SC) (emulsifier and stabilizer)-loaded zein colloidal nanoparticles using an antisolvent method with an average particle size, zeta potential, and PDI of 204.4 ± 12 nm, 38.2 ± 1.4 mV, and ˂0.2, respectively. The results of the antimicrobial evaluation revealed that the amount of thymol present in the films affects their inhibitory activity against *E. coli* and *Salmonella.* ZP_0_ (thymol-to-zein ratios of 0%) and ZP_1_ (thymol-to-zein ratios of 10%) showed no significant action, whereas ZP_2_ (thymol-to-zein ratios of 20%), ZP_3_ (thymol-to-zein ratios of 30%) and ZP_4_ (thymol-to-zein ratios of 40%) showed zone of inhibition ranging from 15.89 ± 0.74 mm to 18.81 ± 0.56 mm against *E. coli* and *Salmonella*. Release rate of thymol from nanoparticle-based films followed two-step biphasic mechanism, with an initial burst effect subsequent to slower release, and the inclusion of zein-SC nanoparticles in the film matrices allowed to maintain thymol release supporting the potential use of developed films in antimicrobial food packaging [163]. Vrouvaki et al. developed *Pistacia lentiscus* EO-encapsulated polymeric nanoparticles composed of poly(lactic acid) (PLA) as the shell material and two surfactants, namely poly(vinyl alcohol) (PLA/PVA-NPs) and lecithin (PLA/LEC-NPs), with a particle size, PDI, and zeta potential of 239.9 nm and 286.1 nm, 0.081, and −29.1 mV, respectively, a using solvent evaporation method. The MIC of EO against *E. coli* and *B. subtilis* was found to be 5 mg/mL and 1.25 mg/mL, respectively. PLA/PVA-NPs tested at 3.4 mg/mL of EO concentration demonstrated prolonged release, supporting their use as carriers for topical applications [164]. Iannitelli et al. prepared carvacrol-loaded PLGA nanocapsules, by means of a solvent displacement approach, to combine the anti-biofilm action of carvacrol with the exceptional delivery characteristics of nanotechnology devices and the excellent biocompatibility of PLGA polymer. The developed nanoparticles had particle sizes, zeta potentials, and PDI values of 209.8 ± 7.2 nm, −18.99 ± 3.01 mV, and 0.260 ± 0.013, respectively. Carvacrol-loaded nanocapsules displayed capacity to change the characteristics of preformed staphylococcal biofilms, with treatment resulting in a significant reduction in the elasticity and mechanical stability of preformed biofilms, enabling antimicrobial agents to penetrate the deep core of bacterial biofilms [165]. Froiio et al. developed sweet orange essential oil (OEO)- and bergamot essential oil (BEO)-loaded-Eudragit RS 100 (EuRS100) NPs of particle sizes ranging from 120 to 150 nm with positive surface charges of 39 to 74 mV using the nanoprecipitation approach. The BEO-NPs and OEO-NPs exhibited encapsulation efficiencies of 28–84% and 48–96%, respectively. Evaluation of antibacterial efficacy revealed a 90% inhibition rate for the developed nanocapsules, which was attributed to a combination of the cationic nature of Eudragit particles and the presence of EOs in the formulation [109]. Qiu et al. developed menthone-encapsulated spherical-shaped starch nanoparticles (SNPs) with particle sizes of 93 to 113 nm from short glucan chains using an in situ nanoprecipitation approach. The developed nanoparticles exhibited an encapsulation efficiency and PDI of 86.6% and 0.156 to 0.262, respectively. The SNPs did not exhibit antimicrobial activity against *S. aureus* and *E. coli*. At the end of 2 h of incubation, the antimicrobial activity of menthone increased noticeably, whereas the activity of M-SNPs increased steadily throughout the test period. SNP-EOs could have potential applications in cosmetics, food preservation, and the control of human pathogenic bacteria [166]. Andriotis et al. synthesized spherical-shaped cross-linked polymeric nanoparticles composed of methyl methacrylate and triethylene glycol dimethacrylate copolymers by means of mini-emulsion polymerization and employed them as matrix-carriers for D-limonene with mean diameters of 0.135 μm. Particles with maximum concentrations (2.53 μg) of D-limonene showed antibacterial activity against *E. coli* (MIC > 100 μL)*, S. aureus, B. subtilis*, and *B. cereus* (MIC < 50 μL) [167]. Wang et al. fabricated eugenol–casein–lysozyme nanoparticles (ECL-NPs) of 151.9 nm and investigated their antibacterial efficiency against gram-positive bacteria. The ECL-NPs exhibited entrapment efficiency, PDI, and zeta potential values of 92.2%, 0.228–0.385, and 34.0–41.4 mV, respectively. The MIC values of ECL-NPs against *S. aureus* and *Bacillus* sp. decreased substantially to 0.16 mg/mL and 0.12 mg/mL, respectively, in comparison to eugenol. The incorporation of lysozyme in the NPs boosted their inhibitory effectiveness against *S. aureus* and *Bacillus* sp., as illustrated by the fact that the MIC was decreased 3.75-fold and 4.16-fold, respectively. Furthermore, fresh fruits treated with ECL-NPs for 15 days showed 100% microbial suppression [168]. Dahlia et al. developed thymol-/carvacrol-loaded polythioether nanoparticles (TCNPs), with mean particle sizes of 147 ± 19 nm and a PDI of 0.387 by means of a one-pot, solvent-free miniemulsion thiol-ene photopolymerization technique. An evaluation of their antimicrobial activity revealed that TCNPs inhibited the growth of antibiotic-resistant *S. aureus* (ZOI = 2 mm), *B. cenocepacia* (ZOI = 3 mm), and *B. subtilis* (ZOI = 6 mm) at a concentration of 25,000 μg/mL. Thymol/carvacrol loading levels in developed NPs are up to 100-fold higher than reported MIC values for tested organisms. Therefore, TCNPs could serve as high capacity reservoirs for slow-release and delivery of thymol/carvacrol- combination payloads that exhibit inhibitory and bactericidal activity (>99.9% kill efficiency at 24 h) against gram-positive (*B. subtilis* and *S. aureus*) and gram-negative (*E. coli* and *B. cenocepacia*) bacteria [108]. Liakos et al. developed cellulose acetate nanocapsules (CANCs) coupled with two antimicrobial agents, lemongrass (LG) essential oil and Cu-ferrite nanoparticles using the solvent/anti-solvent methodology and investigated their synergistic antibacterial activity. Cu-ferrite NPs were immobilized in CA/LG NCs, resulting in a final construct with size of 220–300 nm. CA/LG and CA/LG-Cu-ferrite NCs (50% higher efficacy) were found to exhibit excellent antibacterial properties. Combination of LG and Cu-ferrite NP resulted in significantly potent antimicrobial NCs, as the bacteria were unable to multiply after 20 h of culture [169]. Liakos et al. fabricated LG-loaded cellulose acetate nanocapsules of particle sizes ranging from 95 to 185 nm using the solvent/anti-solvent method. Zeta potential of plain CA-NCs, CA/LG-NCs was found to be <−40 mV and −10 mV, respectively. CA/LG NCs at 50 μL (containing 0.9 μL of LG) caused substantial inhibition of *E. coli* and *S. aureus*. A small amount of LG in CA/LG NCs was capable of inhibiting nearly 70% of *E. coli* and *S. aureus* [170]. Liakos et al. fabricated spherical, bioactive nanocapsules with particle size and PDI of 100–200 nm and 0.09–0.27 by mixing cellulose acetate with peppermint (CA-P-NCs), cinnamon (CA-CN-NCs), and lemongrass (CA-LG-NCs) Eos utilizing the solvent/anti-solvent technique. CA-CN-NCs displayed the lowest MIC values for *C. albicans* (0.031%), *E. coli* (0.06%), *S. aureus* (0.125%), and *P. aeruginosa* (0.25%). The MIC values for CA-P-NCs and CA-LG-NCs ranged from 0.25 to 0.5%. Biofilm’s inhibitory effects were predominantly noticeable in *C. albicans* and *S. aureus* biofilms, with monospecific biofilms having their NCs concentrations reduced to 0.03%. Biofilm’s inhibitory effects in *P. aeruginosa* and *E. coli* biofilms were observed at concentrations greater than 0.125% [171]. Upadhyay et al. synthesized nanoencapsulated *Cananga odorata* EO (CoEO) into chitosan nanoemulsion (CoEO-CsNe) using an ionic-gelation method and investigated their antifungal action against *Aspergillus flavus*. CoEO completely inhibited mycelial growth at 2.0 µL/mL, but *A. flavus* completely stopped AFB1 synthesis at 1.5 µL/mL, and CoEO-CsNe entirely inhibited fungal growth and AFB1 secretion at 1.0 µL/mL and 0.75 µL/mL, respectively. CoEO provided 41.75% and 67.03% protection against fungal contamination at the MIC and 2 MIC doses, respectively, whereas CoEO-CsNe treated seeds provided 83.52% protection at the MIC dose, rising to 91.76% at the 2MIC dose. At both doses investigated, nanoencapsulated CoEO in the form of CoEO-CsNe exceeded unencapsulated CoEO in terms of fungitoxic activity [172]. Flores et al. fabricated tea tree oil nanocapsules (TTO-NC) composed of poly(e-caprolactone) and nanoemulsions (TTO-NE) having mean size of 160–220 nm and PDI of less than 0.25 by spontaneous emulsification and interfacial deposition of the preformed polymer and investigated in onychomycosis model for the antifungal activity. Essential oil (2.37 log CUF mL^−1^), TTO-NE (1.45 log CFU mL^−1^) and TTO-NC (1.0 log CFU mL^−1^) respectively exhibited considerable reduction in cell count after the first week of therapy. TTONC was formulation capable of lowering the initial *T. rubrum* concentration (inoculum concentration) to 1.0 log CFUmL^−1^. TTO-NE and EO significantly increased cell count (0.5 and 1.3 log units, respectively) at the end of 14 days, whereas TTO-NC caused only lesser increase (0.12 units log) [173]. Antonioli et al. developed LG-loaded poly(lactic acid) nanocapsules with a mean diameter, zeta potential and encapsulation efficiency of 96.4 nm, 25.2 ± 0.3 mV, and 99%, respectively. The nanoencapsulated essential oil (EO/NC) showed complete inhibition of mycelial development for *C. acutatum* and *C. gloeosporioides* at a concentration of 0.1% (*v*/*v*), which was four times the concentration of EO (0.025% (*v*/*v*)). However, in an in vivo test using apples, nanoencapsulated lemongrass essential oil outperformed plain essential oil in controlling *C. gloeosporioides*, the most common cause of bitter rot in apples. *C. gloeosporioides* exhibited an average diameter lesion of 28 to 32 mm and ~10 mm in apples treated with pure essential oil and encapsulated essential oil, respectively. Delayed release of active constituents from PLNC containing lemongrass essential oil exhibited necrotic effect and reduced the formation of bitter rot lesions over time, along with necrotic effects of EO terpenoids [174]. Karam et al. fabricated chamomile essential oil (CEO) nanocapsules (NCEO) utilizing N-alkyl chitosan derivatives as polysaccharide-based stabilisers and exhibited average diameter of 801.33 ± 14.74 nm and PDI of 0.310 ± 0.01. CEO demonstrated IC_50_ of 3.33 μg/mL and 14.56 μg/mL for promastigotes and intracellular amastigotes, respectively, whereas NCEO exhibited an IC_50_ of 7.18 μg/mL and 14.29 μg/mL for promastigotes and intracellular amastigotes [175]. Singh et al. developed nanocapsules of chitosan loaded with *Bunium persicum* (Boiss) essential oil (BPEO) as a preservative against fungal and AFB1 contamination of stored masticatories. CS-NPs and CSNPs-BPEO displayed mean particle sizes of 268.6 nm and 291.7 nm, respectively and zeta potentials of +41.3 mV and +33.8 mV, respectively. MIC and MFC of BPEO was found to be 1.2 μL/mL and 3.0 μL/mL, respectively. Nanoencapsulation of BPEO improved its bioefficacy compared to free BPEO, implying that the nanoencapsulated form of BPEO should be used to preserve raw masticatories [29]. Jang et al. developed thyme essential oil-loaded Halloysite nanotubes of 15 nm inner and 50 nm outer diameter, using vacuum pulling methods followed by end-capping or a layer-by-layer surface coating approach for complete encapsulation. During the first 10 days, the packaging paper containing TO-loaded HNT capsules displayed good antibacterial activity against *E. coli*. During the first five days, the wrapping paper was very successful in eradicating *E. coli* [176]. López-Meneses et al. fabricated *Schinus molle* EO (SEO)-loaded chitosan nanoparticles by means of an ionotropic gelation method. The CSNP and CS-SEO-NP exhibited average particle size ranging from 200 to 350 nm and from 225 to 363 nm, with PDI values of 0.35 and 0.74, respectively. The zeta potential for CSNP was +43.8 ± 11.25 mV and it reduced slightly to +40.2 ± 7.5 mV following the addition of SEO. Antifungal evaluation using *A. parasiticus* revealed that CS-SE-NP at a concentration of 500 and 250 μg/mL reduced spore germination rates to 30.9 ± 2.2 and 40.8 ± 5.5; 31.6 ± 0.3 and 9.3 ± 3.7 at the end of 18 h and 24 h, respectively. CSNP at same concentrations produced lower inhibition rates which increased on incorporation of SEO in NPs. Enhancement in antifungal activity was attributed to synergism between SEO and CS in NPs [177]. Yilmaz et al. fabricated spherical chitosan nanoparticles (CNPs) loaded with *Origanum vulgare* essential oil (OEO) with average particle size ranging from 290 to 483 nm using the electrospraying technique. Zeta potential and encapsulation efficiency were found to be in the range of +25.2 to 47.7 mV and 70 to 79.6%, respectively. OEO-CSNPs exhibited fungistatic effects against *Alternaria alternata* AY1, with MIC ranging from 0.02 to 0.005% (*w*/*v*) [178].

### 6.8. Silica Nanoparticles

Silica nanoparticles (SNPs) are of two types: nonporous (solid) silica nanoparticles and mesoporous silica nanoparticles with pore size ranging from 2 to 50 nm. Both types have identical compositions and amorphous structures. However, major differences between the two types are that the MSNs have the porous structure, decreased density, and increased effective surface area. Physical and chemical adsorption into MSNs can load small chemicals, macromolecules, and vaccines, whereas encapsulation and conjugation methods are used to load various drugs into nonporous silica nanoparticles. Pure alkoxysilanes, such as tetraethylorthosilane (TEOS) and substituted alkoxysilanes [R-Si(OR′)_3_] are used to in the synthesis of inorganic and organic silica nanoparticles, respectively [179]. SNPs are cost effective since it could be synthesized using a relatively simple procedure. Furthermore, the morphology, pore size and volume, and particle size can all be altered by altering the parameters during the synthesis [115]. MSNs can encapsulate organic molecules such as EO to effectively limit their volatility and reactivity achieve controlled manner and enhanced the antibacterial and antifungal effects [180].

Das et al. synthesized chamomile EO pickering nanoemulsion (CPe) containing surface modified and stabilized Stöber silica nanoparticles with a mean diameter, PDI value, and zeta potential of 20 nm, 0.01, and 21.3 mV, respectively. CPe was compared to an emulsion stabilised with Tween 80 (CT80) and an ethanolic solution (CEt) in terms of antibacterial activity. The CPe has been shown to have good antibacterial and antifungal activities (MIC_90_) against *E. coli* (2.19 µg/mL), *P. aeruginosa* (1.02 µg/mL), *B. subtilis* (1.13 µg/mL), *S. aureus* (1.06 µg/mL), *S. pyogenes* (2.45 µg/mL), *S. pombe* (1.28 µg/mL), *C. albicans* (2.65 µg/mL), and *C. tropicalis* (1.69 µg/mL), respectively, in comparison to CT80 counterpart. Nanoemulsion exhibited antimicrobial activity on the selected microbes at an average of fourteen-fold less concentration compared with CEt [181]. Das et al. developed surface-modified Stöber silica nanoparticles of 20 nm particle size as the stabilising agent in oil in water type pickering *Artemisia annua* EO nanoemulsions (AEP). Antibacterial activity of AEP on mature *Candida* biofilms was compared to Tween 80 stabilized Artemisia EO emulsion (AET) and ethanolic solution of Artemisia EO (AEE). AEP and AET displayed droplet diameters of 160 ± 2.2 nm and 130 ± 0.9 nm, respectively. The AEP showed significant antibacterial and antifungal activities (MIC_90_) on *Escherichia coli* (1.68 ± 0.72 µg/mL), *Staphylococcus aureus* (1.62 ± 0.37 µg/mL), *B. subtilis* (1.42 ± 0.64 µg/mL), *P. aeruginosa* (1.46 ± 0.22 µg/mL), *S. pyogenes* (3.15 ± 0.16 µg/mL), *S. pombe* (2.01 ± 0.46 µg/mL), *C. albicans* (3.62 ± 0.65 µg/mL), *C. tropicalis* (4.29 ± 0.82 µg/mL), *C. dubliniensis* (3.63 ± 0.57 µg/mL) and *C. krusei* (3.79 ± 0.57 µg/mL), respectively, in comparison to AET. AEP demonstrated superior antibacterial efficacy at an average of twelve-fold less concentration in comparison to AEE [111]. Satary et al. developed lemongrass and clove Eos that were encapsulated into mesoporous silica nanoparticles (LGO-MSNPs and CO-MSNPs) coated with alginate using the sol–gel method to combat *Gaeumannomyces graminis*, causative organism of wheat take all disease. Lemongrass oil exhibited MIC and MFC of 96.8 and 121 mg/L, respectively but these amounts significantly decreased after encapsulation in MSNPs (MIC: 31.76 mg/Land MFC: 39.7 mg/L) however, clove oil showed fungicidal efficacy at MIC and MFC of 137.8 mg/mL, which decreased 46 mg/mL post loading in MSNPs. Treatment of seeds with LGO-MSNPs (74.4%) and CO-MSNPs (71.27%) inhibited *Gaeumannomyces graminis* more effectively than pure EOs. Alginate coated EOs-MSNPs resulted in better disease control than non-alginate EOs-MSNPs. The rate of disease control was as follows: alginate-EOs-MSNPs (LGO and CO: 84%) > EOs-MSNPs (LGO: 74.44%, CO: 71.27%) > pure EOs-MSNPs (LGO: 74.44%, CO: 71.27%) (LGO: 57.44%, CO: 48.93%). These findings implied that alginate could be a good option for coating seeds with natural fungicidal chemicals that aid in seed maintenance, plant growth, and disease prevention [182]. Jin et al. fabricated pepper fragrant EO (PFEO) into MSNs (MCM-41). EONs of an average size and zeta potentials of 717 ± 13.38 nm and 43.90 ± 0.67 mV, respectively, to boost the application of PFEO in the food preservative. PFEO inhibited *E. coli, S. enteric, S. aureus*, and *L. monocytogenes* growth by around 0.396 ± 0.014–0.840 ± 0.025, 0.263 ± 0.021–0.846 ± 0.016, 0.081 ± 0.071–0.83 ± 0.019, and 0.152 ± 0.028–0.99 ± 0.005, respectively. The essential oil nanoparticles demonstrated a total inhibitory concentration (MBC) of 8 mg/mL against *Escherichia coli* and *S. enterica* cells and 20 mg/mL against *S. aureus*. The gradual increase in the release EONs obtained during 48 h incubation proved its potential for food preservation [183]. Ellahi et al. developed polypropylene film coated with silica nanoparticles and *Pistacia atlantica* tree gum essential oil (GEO). An evaluation of the antibacterial activity of the developed packaging film against *S. aureus, S. enterica, E. coli*, and *L. monocytogenes* revealed that 0.001 g silica nanoparticle exhibited a higher inhibitory effect, proving its suitability as an antimicrobial pack. A package containing unloaded silica nanoparticles exhibited no antibacterial effects. GEO was released more quickly from a film devoid of NPs, thus reducing the shelf life of packaged foods. The shelf life of milk was prolonged by 35 days due to the developed antibacterial active packaging [184]. Zhu et al. fabricated a food packaging film using polylactic acid and mesoporous silica nanoparticles loaded with clove essential oil (PMC films) by means of a solvent volatilization method. Various bacteria commonly occurring in *Agaricus bisporus* (white button mushrooms) packages during storage include mesophilic bacteria, psychrophilic bacteria and *Pseudomonas* sps. The log_10_ CFU/mL values of developed packaging films were lower than those of plain PLA films. Due to the addition of CEO, the log_10_ CFU/mL values decreased, indicating that PMC films effectively suppressed the development of microbes. As a result, PMC films could be utilised to preserve the quality of white button mushrooms and extend their shelf life [185].

### 6.9. Nanoemulsion-Based Nanoparticle Candies

Karimi et al. developed thymol, cardamom essential oil, and *L. plantarum* cell-free supernatant nanoparticle candies (nT, nCEO, nS) and investigated their inhibitory activity against *S. mutans* causing tooth decay. The mean diameters and zeta potentials of nCEO, nT, and nS were 74.7 nm and −12.1 mV, 76.6 nm and −22.3 mV, and 92 nm and −41.2 mV, respectively. Thymol displayed the greatest inhibitory effect against *S. mutans* with an MIC of 0.01 mg/mL. The MICs of supernatant and nS were 0.06 mg/mL and 0.01 mg/mL, respectively, and thus, nS exhibited significantly better antibacterial activity against *S. mutans* than free S. The MICs for CEO and nCEO against *S. mutans* were found to be 0.03 and 0.05 mg/mL, respectively. The inhibitory effect of nCEO was significantly higher than that of free CEO. Thus, nS was proved to be the best treatment for tooth decay in this study [186].

Nine different types of nanocarriers discussed in this review had the desired shape and size and were capable of achieving controlled release, improved stability, and prolonged antimicrobial activity of encapsulated or adsorbed EOs. Among all the reported NPs with desirable characteristics, polymeric nanoparticles were extensively explored for the encapsulation of EOs; this was attributed to their unique features such as biocompatibility, biodegradability, hydrophilicity, stability, safety, and nontoxicity [187]. A porous or cavity-like structure formed by polymers provides structural support for encapsulating EOs. Nanocapsules have benefits including larger drug loadings (and less polymer), better active molecule protection, and reduced burst release. Amongst the polymeric nanoparticles, chitosan nanoparticles are widely used due to their excellent encapsulation efficiency and prolonged release of EOs. A comparative analysis of the enhancement in the antibacterial activity of EO-loaded nanoparticles was carried out. Peppermint oil was encapsulated in three different nanoparticles, namely hydroxyapatite NP, magnetite NP, and polymeric NP. Among these nanoparticles, EO encapsulated in polymeric nanoparticles demonstrated enhancement in antimicrobial activity. Clove oil was encapsulated into lipid NP, polymeric NP, and silica NP, and better antimicrobial activity was seen in lipid nanoparticles. Tea tree oil demonstrated enhanced antibacterial activity when encapsulated into lipid nanoparticles. EO constituent eugenol was encapsulated into ethosomal NP and showed better antimicrobial activity as compared to polymeric and magnetite NPs. Thymol demonstrated better zone of inhibition when encapsulated in polythioether (polymeric NP). Some EO-encapsulated nanoparticles showed prolonged antimicrobial activity rather than enhanced effect. Such nanoparticles helped to increase the shelf-life food products while controlling the bacterial growth. In addition, some of the investigated nanoparticles including metal and magnetite nanoparticles having inherent antimicrobial property demonstrated synergism with co-loaded EOs.

## 7. Synergistic Action of EO and Nanoparticles

Synergism in the antimicrobial activity of EOs and NPs in combination is widely investigated. Dhyea and Jallil biosynthesised TiO_2_-NPs using *Allium cepa* and *Capsicum annum* and investigated the antimicrobial activities of NPs and tried to enhance these activities by means of *Eugenia caryophyllata* essential oil in combination therapies. The average diameter of NPs synthesised were in the range of 89.1–103.60 nm. It was found that NPs displayed antimicrobial action against *K. pneumonia, S. aureus, S. epidermidis, E. coli,* and *C. albicans* when either NP (0.01–1 mg/mL) or EO (3.125–50%) are used alone. Five combinations of NPs (*Allium cepa*) and Eos were prepared and exhibited four synergistic combination points in *S. aureus*, one synergistic combination point in *E. coli*, and three synergistic combination points in *C. albicans*, and an antagonist effect on *K. pneumonia* and *S. epidermidis* was also observed; furthermore, the five combination therapies of NPs (*C. annum*) with EO exhibited one synergistic point each for *K. pneumonia, S aureus*, and *S. epidermidis*, and four synergistic points each for *C. albicans* and *E. coli* [188]. Scandorieiro et al. developed oregano essential oil (OEO) and biologically synthesised silver nanoparticles (bio-AgNP) using *Fusarium oxysporum* and evaluated the antibacterial activity of a two-drug combination. OEO (MIC value 0.526 ± 0.130 mg/mL) and bio-AgNP (MIC range 62.5 to 250 μM) in combination showed considerably lower MIC values against S. aureus (0.149 mg/mL and 62.5 µM for OEO and bio-AgNP, respectively) and *E. coli* (0.298 mg/mL and 15.62 µM, respectively) than when used separately, indicating that the two compounds have synergistic or additive antibacterial activity against gram-positive and gram-negative bacteria, including multidrug-resistant strains [189]. Biasi-Garbin et al. investigated the effect of eugenol on *S. agalactiae* (group B *streptococci* (GBS)) isolated from colonized women, alone and in combination with silver nanoparticles generated by *Fusarium oxysporum* (AgNPbio). Eugenol displayed MIC/MBC values ranging from 0.125 to 0.5% against the GBS strains. AgNPbio alone inhibited the GBS strains at a concentration of 125 μM. It was revealed that two chemicals when used in combination, the MIC values of eugenol and AgNPbio reduced by 4–8 fold and 4–256 fold, respectively. The combination of eugenol and AgNPbio produced a substantial synergistic effect, lowering the minimum inhibitory concentration of both compounds significantly [190]. Ghosh et al. developed AgNPs and cinnamaldehyde together and evaluated the extent of synergy. The results demonstrated that when nanoparticles were used in the presence of cinnamaldehyde, their activity was considerably increased. The combination showed significant decrease in *B. cereus* and *C. perfringens*. At the synergy point for *C. perfringens*, the inhibitory concentration of AgNPs was reduced from 612.5 ng/mL alone to 153.1 ng/mL in combination, while the MIC of cinnamaldehyde was reduced from 0.065 mg/mL alone to 0.016 mg/mL in combination. At synergy point in *B. cereus*, the inhibitory concentration of AgNPs was reduced from 1225 ng/mL alone to 306.3 ng/mL, while the MIC of cinnamaldehyde was reduced from 0.328 mg/mL alone to 0.082 mg/mL in combination The combination demonstrated at least an additive or near-synergistic impact as MIC value of AgNPs and cinnamaldehyde reduced significantly for *B. cereus, C. perfringens, M. luteus, P. aeruginosa*, and all other strains tested [191]. Sheikholeslami et al. fabricated silver nanoparticles (AgNPs) and investigated antibacterial activity of AgNPs alone and in conjunction with *Zataria multiflora* essential oil and methanolic extract. MIC and MBC values of Ag-NPs against *S. aureus, MRSA, S. epidermidis* and *P. aeruginosa* were observed in the range of 15.625–500 µg/mL, and those of EO and methanolic extract were in the range of 1.56–100 mg/mL. Results suggested that combination of silver nanoparticles with essential oil against *S. epidermidis* and *S. aureus* caused an additive effect, as defined by fractional inhibitory concentrations values of 0.6248 and 1, respectively, while combination of silver nanoparticles and plant extracts showed an additive effect against *S. epidermidis*, as measured by FIC value of 1 [192]. Abd El-Aziz et al. developed cinnamon oil, silver nanoparticles (AgNPs), and studied the antimicrobial and antibiofilm potency of their combination against multidrug-resistant (MDR) *S. agalactiae* collected from clinical bovine mastitis in Egypt. Cinnamon oil exhibited strong antimicrobial (MICs ≤ 0.063 μg/mL) and antibiofilm (MBIC_50_ = 4 μg/mL) effects against planktonic and biofilms of *S. agalactiae* isolates, respectively. AgNPs, on the other hand, displayed reasonable antimicrobial (MICs ≤ 16 μg/mL) and relatively low antibiofilm (MBIC_50_ = 64 μg/mL) properties against investigated isolates. The MICs of cinnamon oil and AgNPs were significantly decreased 4 to 512 fold and 32 to 128 fold, respectively, when the two compounds were combined, indicating that the combined activity of antimicrobial agents against planktonic *S. agalactiae* isolates was greater than the sum of their independent activity [193]. Cinteza et al. designed AgNPs stabilized with chitosan as capping agent which ensured a stable, nontoxic metallic Ag nanostructure. MIC of TO against *E. coli, P. aeruginosa, S. aureus* and *C. albicans* was found to be 0.25%, 0.25%, 0.50% and 0.50% while MIC of CO was found to be 0.25%, 0.13%, 0.13% and 0.13% respectively. The MIC of AgNPs and AgNP-CS against the screened microorganisms was found to be 1.56 ppm, 1.56 ppm, 3.13 ppm, and 0.78 ppm, respectively, for both. The chitosan-stabilized AgNPs showed a strong inhibitory effect on the growth of all bacterial and fungal strains examined. The minimum inhibitory concentration of AgNPs was reduced by one or two orders of magnitude when essential oils from thyme and clove, natural products with established antibacterial characteristics, were added to colloidal dispersions indicating that antibacterial efficacy of AgNP could boost when paired with clove and thyme EOs [194].

## 8. Conclusions and Future Perspectives

Essential oils have been widely explored as potential sources of antimicrobial agents in therapies, food preservation and packaging. However their commercial use has been restricted due to issues such as low solubility, solvent toxicity, volatility, and strong organoleptic flavor. Nanoparticles are viable candidate for the production of EO-based antimicrobial nanosystems due to their biocompatibility, biodegradability, nontoxicity, and target selectivity. Researchers have explored nanotechnology-based versatile encapsulation strategies for EOs such as solid lipid nanoparticles, inorganic nanoparticles, polymeric nanoparticles, nanogels, liposomes, silica nanoparticles and metallic nanoparticles to mask their undesirable attributes and enhance biological activities.

Commonly explored methods for the preparation of EO-loaded nanoparticles include co-precipitation, high-pressure homogenization, high-sheer homogenization with ultrasonication, ionic gelation, mini-emulsion polymerization, nanoprecipitation, spray drying, the Stöber process, thin film hydration, adsorption, and the vacuum pulling method. The majority of scientific investigations reported in relation to the development of EO-loaded nanoparticles involved co-precipitation and ionic gelation methods. Magnetic nanoparticles and polymeric nanoparticles specifically based on chitosan polymer are most widely investigated, which is attributed to their synergistic effect. Magnetite nanoparticles cause antimicrobial effects through membrane depolarization, the impairment of cell integrity, the disruption of cellular homeostasis, protein coordination, the production of reactive oxygen species, and DNA damage. Chitosan has potent antibacterial activity due to its binding to the negatively charged molecules of the bacterial cell membrane, which leads to enhanced drug permeation. Various applications that have been explored for EO-based nanoparticles include the antimicrobial effect in therapy, biofilm formation, packaging material, and food preservation. Rosemary EO-loaded TiO_2_ nanoparticles, carvacrol-loaded ZnO nanoparticles, geraniol-loaded ZnO nanoparticles, fennel EO-loaded ZnO nanoparticles, thymol-loaded zein nanoparticles, D-Limonene-loaded methyl methacrylate (MMA) and triethylene glycol dimethacrylate (TEGDMA) nanoparticles, thyme EO-loaded halloysite nanotubes, pepper fragrant EO-loaded silica nanoparticles, *Pistacia atlantica* tree gum oil-loaded silica nanoparticles, and Rosemary EO-loaded chitosan-benzoic acid nanogel are used for antimicrobial packaging. Eugenol-loaded ethosome nanoparticles, *Zataria multiflora* EO-loaded chitosan nanoparticles, Guava leaf EO-loaded chitosan nanoparticles, *Thymus capitatus* and *Origanum vulgare* EO-loaded chitosan nanoparticles, Mandarin EO-loaded chitosan nanoparticles, Bergamot and Sweet orange EO-loaded Eudragit nanoparticles, and *Bunium persicum* EO-loaded chitosan nanoparticles are used for food preservation. The most commonly explored essential oil-loaded nanosystems are reported for peppermint oil, lemongrass oil, tea tree oil, clove oil, black cumin oil, etc. Volatile constituents such as eugenol-, thymol-, and carvacrol-loaded nanoparticles are also reported. Various nanoparticulate systems such as liposomes, magnetite nanoparticles, polymeric nanoparticles, lipid nanoparticles, and inorganic nanoparticles were designed for encapsulation of EO constituents. Large numbers of EO-based nanosystems exhibited antimicrobial effects against *S. aureus*, *E. coli*, *K. pneumoniae*, *L. monocytogenes, P. aeruginosa* and *C. albicans.* Several Eos, such as cajuput oil, eucalyptus oil, cumin oil, marjoram oil, Jamaica pepper oil, betel leaf oil, cedarwood oil, ageratum oil, palmarosa oil, Aegle oil, aniseed oil, etc., are still unexplored in the development of EO-based antimicrobial nanosystems. α-phellandrene, carvone, farnesene, borneol, p-cymene, camphene, α-thujene, fenchone, etc. are some of the essential oil constituents that can be formulated into nanoparticles.

## Figures and Tables

**Table 1:** Major chemical components of essential oils and their antimicrobial activities.

Biological Source of Essential Oils	Part	Antimicrobial Activities	Major Chemical Components	Mechanism of Action	References
*Bunium persicum*	Seeds	*L. monocytogenes*, *Listeria grayi andAspergillusflavus*	γ-Terpinene, 1-phellandrene, γ-terpene, cuminaldehyde	Cell membrane disruption and cytolytic leakage Swelling and reduction in membrane function	[10,29,30,31]
*Cananga odorata*	Flower	Hepatitis B virus (HBV), *Bacillus. subtilis, E. coli, S. typhi, Shigella shiga, Streptococcus-β-haemolyticus* and *A. flavus*	Linalool, *β*-caryophyllene	Disruption of cell membrane integrity Induces apoptosis via nuclear condensation and fragmentation pathways including disruption of mitochondrial membrane potential	[13,32,33,34]
*Carum copticum*	Seeds	*S. aureus, Staphylococcus epidermidis, Bacillus cereus, E. coli, S. typhimurium, Proteus vulgaris*	Thymol, γ-Terpinene, ρ-cymene	Depolarization of the cytoplasmic membrane and disruption of cell membrane integrity and decrease intracellular ATP levels	[12,35,36]
*Cinnamomum zeylanicum*	Bark	*Borrelia burgdorferi, E. coli., S. aureus, and P. aeruginosa*	Carvacrol	Depolarization of the cytoplasmic membrane and disruption of cell membrane integrity	[12,37,38]
*Citrus bergamia*	Peel	*Campylobacter jejuni, E. coli, L. monocytogenes, B. cereus,* and *S. aureus*	Linalool, Citral, Linalyl acetate	Disruption of cell membrane integrity Induction of changes in ATP concentration, cell membrane hyperpolarization, and reduction in cytoplasmic pH	[39,40]
*Citrus reticulata*	Peel	*S. aureus, E. coli, Penicillium italicum* and *Penicillium. digitatum*	Limonene and γ-Terpinene	Cell membrane disruption and cytolytic leakage	[41]
*Cymbopogon citratus*	Leaves	HSV-1, HSV-2, *S. aureus*, *E. coli* and *Gaeumannomyces graminis*	Citral	Induction of changes in ATP concentration, cell membrane hyperpolarization, and reduction in cytoplasmic pH	[42,43]
*Eugenia caryophyllata*	Flower buds	*B. cereus, S. typhimurium and E. coli*	Eugenol, β-caryophyllene	Cell membrane disruption and cytolytic leakage Induces apoptosis via nuclear condensation and fragmentation pathways including disruption of mitochondrial membrane potential	[13,31,44]
*Eucalyptus globulus*	Leaves	*S. aureus and S. pyogenes*	1,8-cineol α-pinene	Disruption of cell membrane integrity and cytolytic leakage	[45]
*Foeniculum vulgare*	Seeds and Leaves	*S. aureus, E. coli, and A. flavus*	Anethole	Disruption of cell membrane integrity	[46,47]
*Homalomena pineodora*	Leaves	*B. cereus, B. subtilis, S. aureus, MRSA, E. coli, Proteus mirabilis, Yersinia sp., K. pneumoniae, Shigella boydii, S. typhimurium, Acinetobacter anitratus, P. aeruginosa, Candida albicans and Candida utilis*	2-octylcyclopentanone	Cell membrane disruption and cytolytic leakage	[48]
*Lavandula angustifolia Sevastopolis*	Whole plant	MRSA, *S. aureus* and *E. coli*	Linalool, Borneol, Camphor	Disruption of cell membrane integrity and cytolytic leakage	[11,49]
*Lippia sidoides*	Leaves	*Stegomyia aegypti* larvae	Thymol	Depolarization of the cytoplasmic membrane and disruption of cell membrane integrity	[12,50]
*Matricaria chamomilla*	Fresh or dried flower heads	*Leishmania amazonensis,E. coli, P. aeruginosa, B. subtilis, S. aureus, S. pyogenes, Schizosaccharomyces pombe, C. albicans* and *Candida tropicalis*	α-Bisabolol	Cell membrane disruption and cytolytic leakage	[51]
*Melaleuca alternifolia*	Leaves	*S. aureus, E. coli, L. monocytogenes, C. albicans, P. aeruginosa and A. niger*	Terpinen-4-ol	Cell membrane disruption and cytolytic leakage	[52,53]
*Mentha piperita*	Leaves	*C. albicans, C. tropicalis, Pichia anomala and* *Saccharomyces* *cerevisiae*	Menthol, Menthone	Depolarization of the cytoplasmic membrane and disruption of cell membrane integrity	[54]
*Nigella sativa*	Seeds	*S. aureus and Vibrio harveyii*	Thymoquinone	Apoptosis by production of reactive oxygen species	[55,56]
*Ocimum basilicum*	Whole plant	*C. albicans, S. aureus*	Linalool	Disruption of cell membrane integrity and cytolytic leakage	[34,40]
*Origanum vulgare*	Leaves	*Trichophyton tonsurans, Trichophyton violaceum, Trichophyton floccosum, T mentagrophytes*	Carvacrol, Thymol	Depolarization of the cytoplasmic membrane and disruption of cell membrane integrity	[12,57]
*Pistacia atlantica*	Gum	*S. aureus, S. enterica, E. coli and L. monocytogenes*	α-Thujene, α-Pinene, Camphorene, Sabinene, β-Pinene, ∆3-Carene, Limonene	Disruption of cell membrane integrity and cytolytic leakage	[58,59]
*Pistacia lentiscus*	Resin	*E. coli and B. subtilis*	α-Pinene, β-Pinene, β-myrcene, Linalool, *trans*-Caryophyllene and Camphene	Disruption of cell membrane integrity and cytolytic leakage	[59,60]
*Psidium guajava*	Leaves	*S. aureus, Salmonella spp. and E. coli*	β- caryophyllene	Induction of apoptosis via nuclear condensation and fragmentation pathways including disruption of mitochondrial membrane	[13,61]
*Punica granatum*	Seeds	*S. epidermidis*	Punicalagin, punicalin	Cell membrane disruption and cytolytic leakage	[62,63,64]
*Rosmarinus officinalis*	Leaves	*C. albicans, C. tropicalis*	1,8-Cineole, camphor	Disruption of cell membrane integrity and cytolytic leakage	[65,66]
*Satureja hortensis*	Leaves	*S. aureus, Corynebacterium glutamicum, P. aeruginosa and E. coli, and C. albicans*	Carvacrol, Thymol	Depolarization of the cytoplasmic membrane and disruption of cell membrane integrity	[12,67]
*Syzygium aromaticum*	Floral bud	*E. coli, S. aureus, S. typhi, P. aeruginosa, B. cereus, L. monocytogenes*	Eugenol, eugenyl acetate	Cell membrane disruption and cytolytic leakage	[31,68]
*Thymus vulgaris*	Leaves	*M. furfur, C. albican, C. tropicalis, Candida glabrata, Candida kefyr and Candida guillermondii, S. aureus, S. pyogenes and E. coli*	Thymol, p-cymene, Carvacrol	Depolarization of the cytoplasmic membrane and disruption of cell membrane integrity	[12,69,70]
*Zataria multiflora*	Aerial parts	*S. aureus*, MRSA, *S. epidermidis* and *P. aeruginosa*	Carvacrol, Thymol, p-cymene	Depolarization of the cytoplasmic membrane and disruption of cell membrane integrity	[12,71]

**Table 2:** Antibiotics in combination with Essential oils and their interactions.

Antibiotics	Essential Oils/Essential Oil Constituents	* FICI	Organisms	Interaction	Reference
Amoxicillin, Ciprofloxacin	Ajowan oil Thymol	0.36–1	*P. aeruginosa, S. aureus* and *S. pneumoniae*	Synergism—EO/thymol with amoxicillin against MRSA; EO with ciprofloxacin against *P. aeruginosa, S. aureus* and *S. pneumoniae;* Thymol with ciprofloxacin against *P. aeruginosa* and *S. pneumoniae*	[73]
Cefepime	Rosemary oil	-	*P. aeruginosa*	Synergism	[74]
Ciprofloxacin Fluconazole	*Thymus atlanticus*	0.25–0.50	*Bacillus subtilis*, *Micrococcus luteus*, *Staphylococcus aureus, Pseudomonas aeruginosa*, *Escherichia coli*, *K. pneumoniae* and *Candida parapsilosis,* *Candida albicans, Candida glabrata, Candida krusei*	Synergism	[75]
Ciprofloxacin Fluconazole	*Linaria ventricosa*	0.26 to 0.50	*E. coli, C. albicans* and *C. glabrata*	Synergism	[76]
Doxycycline	Carvacrol, eugenol and cinnamaldehyde	0.7–1.3	*Acinetobacter baumannii* *K. pneumoniae* *E. coli* *P. aeruginosa*	Additive or indifferent inhibitory activity Synergistic bactericidal activity	[77]
Fluconazole Amphotericin B	*T. satureioides* *T. pallidus* *A. leucotrichus* *T. leptobotrys* *O. compactum* *A. herba alba*	0.25–0.31	*C. albicans* *C. glabrata* *C. krusei* *C. parapsilosis*	Synergism	[78]
Fluconazole Amphotericin B	*Citrus aurantium*	0.36 and 0.24	*Candida albicans*	Synergism	[79]
Fluconazole, Ciprofloxacin Vancomycin	*Laurus nobilis* *Prunus armeniaca*	0.258–0.75	*M. luteus,* *S. aureus, B. subtilis, E. coli, P. aeruginosa, K. pneumoniae and* *C. parapsilosis,* *Candida albicans, Candida glabrata, Candida krusei*	Synergism	[80]
Fluconazole, Econazole, Ketoconazole Itraconazole	Melaleuca leucadendra	0.35–0.46	*C. albicans*	Synergism	[81]
Octenidine dihydrochloride	Lavender	0.11–0.26	MRSA	Synergism	[82]
Oxacillin, Amoxicillin, Gentamicin, Ciprofloxacin, Tetracycline, Erythromycin, Clindamycin	coriander oil	0.25–1	MRSA *S. epidermidis* *P. aeruginosa* *E. coli*	Synergism—coriander oil with amoxicillin, gentamicin, oxacillin and tetracycline against MRSA; coriander oil with gentamicin against *P. aeruginosa;* coriander oil with erythromycin and tetracycline against *E. coli* Additive—coriander oil with amoxicillin and clindamycin against MRSA; coriander oil with gentamicin and ciprofloxacin against *E. coli*	[83]
Polymyxin B	*Cinnamomum cassia*	0.006	carbapenemase-producing *Klebsiella pneumoniae* and *Serratia marcescens*	Synergism	[84]
Sarafloxacin, Levofloxacin, Polymycin, Lincomycin, Amoxicillin, Ceftiofur, Ceftriaxone, Maquindox, Florfenicol, Doxycycline, Kanamycin	Oregano	0.375–1.5	*E. coli*	Synergism—oregano oil with Sarafloxacin, Levofloxacin, Maquindox, Florfenicol, Doxycycline Additive—oregano oil with Polymycin, Lincomycin, Amoxicillin, Ceftiofur, Ceftriaxone Independent—oregano oil with Kanamycin	[85]
Streptomycin Ampicillin Chloramphenicol	*Cinnamomum cassia*	0.38–0.125	*E. coli*, *S. aureus*, and *P. aeruginosa*	Synergism—EO with chloramphenicol against *E. coli* and *S. aureus* Additive—EO with Streptomycin and Ampicillin against *E. coli, S. aureus* and *P. aeruginosa*	[86]
β-lactam antibiotics (methicillin, penicillin G)	1,8-cineole, eugenol, carvacrol, linalool, linalyl acetate, *trans*-anethole, thymol, menthone, menthol, β-caryophyllene	0.2–5.0	MSRA	Synergism—linalyl acetate with methicillin and 1,8-cineole with penicillin G Additive—linalyl acetate with penicillin G Antagonism—methicillin with thymol and methicillin with menthone	[87]

* Fractional Inhibitory Concentration Index.

**Table 3:** Clinical trial data of some Essential oils.

Sr. No.	Study Title	Condition	Interventions	Study Design		Phase	Location	Status	Outcome Measurement	Reference
1.	Effect of a Medicated Topical Therapy, Petrolatum, and No Treatment on Nocturnal Cough	Respiratory tract Diseases	Other: Ointment containing camphor, eucalyptus oil, and menthol One time use Other: Petroleum jelly One time use	**Study Type:**	Interventional (Clinical Trial)	-	United States, Pennsylvania	Complete	Subjective assessment of cough and congestion symptoms (Time Frame: 24 h)	[88]
				**Actual Enrollment:**	143 participants					
				**Allocation:**	Randomized					
				**Intervention Model:**	Parallel Assignment					
				**Masking:**	Double (Participant, Investigator)					
				**Primary Purpose:**	Treatment					
2.	Treatment of Acute Rhino-Sinusitis with Essential Oils of Aromatic Plants	Rhino-Sinusitis	Dietary Supplement: mixture of aromatic essential oils. 1% of mixture containing aromatic essential oils of Eucalyptus citriodora, Eucalyptus globulus, Mentha piperita, Origanum syriacum, and Rosmarinus Officinalis, spraying to the nose. Dietary Supplement: placebo 0.1% of Lemon VIP (Florasynth, Israel), spraying to the nose.	**Study Type:**	Interventional (Clinical Trial)	I and II	Israel	Complete	To demonstrate a relief in the nasal obstruction within the first 20 min after first administration of treatment with the spray. (Time Frame: 20 min) To demonstrate a reduction in a defined symptoms sum score based on symptoms and signs comparing baseline therapy from the beginning to the end of 3 days treatment. (Time Frame: 3 days)	[89]
				**Actual Enrollment:**	14 participants					
				**Allocation:**	Randomized					
				**Intervention Model:**	Parallel Assignment					
				**Masking:**	Double (Participant, Investigator)					
				**Primary Purpose:**	Treatment					
3.	Treatment of Acute Tracheitis and Laryngitis With Essential Oils of Aromatic Plants	Viral Laryngitis Viral Tracheitis	Dietary Supplement: mixture of aromatic essential oils. 3% of mixture containing aromatic essential oils of Eucalyptus citriodora, Eucalyptus globulus, Mentha piperita, Origanum syriacum, and Rosmarinus Officinalis, spraying to the larynx. Dietary Supplement: placebo 0.1% of Lemon VIP (Florasynth, Israel), spraying to the larynx	**Study Type:**	Interventional (Clinical Trial)	I and II	Israel	complete	To demonstrate a cough or hoarseness relief within the first 20 min after first administration of treatment with the spray. (Time Frame: 20 min) To demonstrate a reduction in a defined symptoms sum score based on symptoms and signs comparing baseline therapy from the beginning to the end of 3 days treatment. (Time Frame: 3 days)	[90]
				**Actual Enrollment:**	29 participants					
				**Allocation:**	Randomized					
				**Intervention Model:**	Parallel Assignment					
				**Masking:**	Double (Participant, Investigator)					
				**Primary Purpose:**	Treatment					
4.	Anosmia Rehabilitation in Patients Post Coronavirus Disease (COVID 19)	Olfactory Disorder	Other: Olfactory retraining Olfactory retraining Olfactory training is performed by exposing patients twice daily to essential oils with four specific odors, present in glass jars with soaked cotton pads: phenyl ethyl alcohol, rose; eucalyptol, eucalyptus; citronellal, lemon; eugenol, cloves. Drug: corticosteroid nasal irrigation Other: smell household Items Other: Nasal Irrigation	**Study Type:**	Interventional (Clinical Trial)	IV	Canada, Ontario	With-drawn	Change from Baseline Snap and Sniff Threshold Test and Smell Identification Test (SIT) at 3 months (Time Frame: 3 and 6 months) Score from the Snap and Sniff Olfactory Test results and Smell Identification test results.	[91]
				**Actual Enrollment:**	0 participants					
				**Allocation:**	Randomized					
				**Intervention Model:**	Parallel Assignment					
				**Masking:**	None (Open Label)					
				**Primary Purpose:**	Treatment					
5.	A Randomized Study to Evaluate the Efficacy of Herbal Ingredients Combined With a Carrier System (Phytonail) Compared With Amorolfine 5% Nail Lacquer (Loceryl) in the Treatment of Toenail Onychomycosis	Onychomycosis	Drug: Phytonail Other Name: herbal ingredients combined with a carrier system (Phytonail) Drug: Loceryl Other Name: amorolfine 5% nail lacquer (Loceryl)	**Study Type:**	Interventional (Clinical Trial)	-	Taiwan	Unknown	Mycological cure (Time Frame: At week 16)	[92]
				**Estimated Enrollment:**	72 participants					
				**Allocation:**	Randomized					
				**Intervention Model:**	Parallel Assignment					
				**Masking:**	None (Open Label)					
				**Primary Purpose:**	Treatment					
6.	Omega-3, Nigella Sativa, Indian Costus, Quinine, Anise Seed, Deglycyrrhizinated Licorice, Artemisinin, Febrifugine on Immunity of Patients With (COVID-19)	Covid19 Immunodeficiency	Drug: Omega 3/Nigella Sativa Oil Drug: Omega 3/Nigella Sativa Oil/Indian Costus Drug: Omega 3/Nigella Sativa Oil/Quinine pills Drug: Omega 3/Nigella Sativa Oil/Anise seed capsule Drug: Omega 3/Nigella Sativa Oil/Deglycyrrhizinated Licorice Drug: Active Comparator	**Study Type:**	Interventional (Clinical Trial)	II and III	Saudi Arabia	Recruiting	Clinical improvement (Time Frame: 30 Days) Time to Clinical recovery Recovery rate from positive to negative swaps (Time Frame: 14 Days)	[93]
				**Estimated Enrollment:**	200 participants					
				**Allocation:**	Randomized					
				**Intervention Model:**	Sequential Assignment					
				**Masking:**	Double (Participant, Care Provider)					
				**Primary Purpose:**	Treatment					
7.	Use of Vagitories based on St. John’s Wort, Tea Tree Oil and Shepherd’s Purse in the Treatment of Vaginal Inflammation	Non-Specific Vaginitis	Drug: Shepherd’s Purse extractum oleosum vagitories Drug: Tea tree vagitories Drug: Hyperici extractum oleosum vagitories Drug: Vagitories—Probiotic	**Study Type:**	Interventional (Clinical Trial)	IV	Bosnia and Herzegovina	Complete	Change in objective symptoms of non-specific vaginitis, assessed by gynecological examination (Time Frame: 1 day after treatment completion)	[94]
				**Actual Enrollment:**	210 participants					
				**Allocation:**	Randomized					
				**Intervention Model:**	Parallel Assignment					
				**Masking:**	None (Open Label)					
				**Primary Purpose:**	Treatment					
8.	Efficacy of a Plaque Disclosing Toothpaste on Home Oral Hygiene Procedures	Chronic Gingivitis, Plaque Induced	Other: Colgate toothpaste fluoridated Other: Shoplaq toothpaste Active Ingredient -Sodium Monofluorophosphate 1000 PPM Ingredients -Precipitated Calcium Carbonate, Sorbitol, Glycerin, Precipitated Silica, Sodium Carboxy Methyl Cellulose, Sodium Benzoate, DM Water, Colour CI-45410, Holy Basil Oil, Neem Oil, Citrus Oil, Thymol Oi, Clove Oil, Piper Betel Leaf Oil, Tea Tree Oil, Eucalyptus Oil, Peppermint Oil, Spearmint Oil. Dye containing tooth paste for disclosing plaque and efficient brushing for better oral health.	**Study Type:**	Interventional (Clinical Trial)	-	Malaysia	Unknown	Plaque removal efficacy of a disclosing toothpaste (Time Frame: from Baseline to 1 year)	[95]
				**Estimated Enrollment:**	50 participants					
				**Allocation:**	Randomized					
				**Intervention Model:**	Parallel Assignment					
				**Intervention Model Description:**	interventional preventive trial					
				**Masking:**	Double (Care Provider, Outcomes Assessor)					
				**Masking Description:**	Toothpaste tubes will be masked so the care provider would not know which tube he/she allocating to the participants as well the outcome assessor would be masked from both groups (test and control) so data is assessed unbiased.					
				**Primary Purpose:**	Prevention					

## Data Availability

Not applicable.
